# 17th European College of Equine Internal Medicine (ECEIM) Congress Copenhagen, Denmark 14‐16 November 2024

**DOI:** 10.1111/jvim.17292

**Published:** 2025-01-21

*The European College of Equine Internal Medicine (ECEIM) Congress and the Journal of Veterinary Internal Medicine (JVIM) are not responsible for the content or dosage recommendations in the abstracts. The abstracts are not peer‐reviewed before publication. The opinions expressed in the abstracts are those of the author(s) and may not represent the views or position of the ECEIM. The authors are solely responsible for the content of the abstracts*.

**ORAL ABSTRACTS**

- Pharmacokinetics of co‐administration of misoprostol and sucralfate in adult horses
- Comparing the inter‐ and intra‐rater reliability of two scoring systems for equine gastric glandular disease
- Pituitary gland abscessation after maxillary cheek tooth extraction in three horses
- The seasonality of insulin concentrations in horses
- Retrospective case series on short‐term responses and lipoprotein profiles following treatment with dapagliflozin or ertugliflozin in horses with insulin dysregulation
- A high‐protein meal is associated with increased glucose‐dependent insulinotropic polypeptide secretion in insulin‐dysregulated horses
- Preliminary results on the measurement of beta‐endorphins in horses with pituitary pars intermedia dysfunction
- Vena contracta and proximal isovelocity surface area as potential echocardiographic measurements for severity grading of mitral valve regurgitation
- Development and application of the EFFE (Ecographic Fast Foal Evaluation) protocol: a pilot study
- Neuronal and astroglial dysfunction in critically ill neonatal foals
- Biomarkers of brain injury in foals with neonatal maladjustment syndrome
- Equine corona virus infections in adult horses: investigating seroconversion and fecal shedding patterns
- Seroprevalence of West Nile, Usutu, and Tick borne encephalitis virus in equids from the Southwest of France in 2023
- Comparison of the long‐term humoral immune response against West Nile virus in clinically and inapparently infected horses
- Sequencing bacterial cell‐free‐DNA to detect pathogenic bacteria in the blood of equine neonates; a pilot study
- Nosocomial syndrome surveillance in an equine veterinary teaching hospital: prevalence and feasibility
- Identifying the origin of left atrial ectopy, including pulmonary veins, via multiple catheter recording in the right heart
- Metformin mitigates atrial remodeling in horses with induced chronic atrial fibrillation
- Heart rate variability based upon P‐wave indices in horses: an exploratory study
- Effect of steamed hay on airway inflammation in horses with severe equine asthma
- Dust exposure and pulmonary inflammation in mild asthmatic horses fed pelleted or round bale hay: A pilot study
- Characterization of renal lipidosis in equids: A postmortem case‐control study (2008‐2022)
- Exploring paraoxonase‐1 as a marker of inflammation and oxidative stress in horses with colitis
- Evaluation of biomarkers (BIOs) in healthy and colic horses: correlation with systemic inflammatory response syndrome (SIRS) status and outcome
- Measurement of thymidine kinase‐1 activity in serum and body cavity fluids in horses with lymphoma, inflammatory bowel disease and other non‐inflammatory gastrointestinal disorders
- Histological and immunological comparison of gastrointestinal biopsies with their respective full‐thickness tissue counterpart in horses
- Genome‐wide association study introduced novel genomic loci of insect bite hypersensitivity in Finnhorses

**POSTER ABSTRACTS**

- Longitudinal changes in fecal short chain fatty acids during hospitalization in horses with different types of colic and their association with survival
- Fungal gastric lesions: morphological, histopathological and microbiological features in three horses
- Clinical, clinicopathological, pathological and genetic findings in six Franches‐Montagnes foals with suspected hypertriglyceridemia‐induced pancreatitis
- Association of equine squamous and glandular gastric disease with dental status in 54 horses
- Antimicrobial treatment approaches to horses with acute diarrhea admitted to referral institutions
- Does equine asthma predispose horses to premature complexes during exercise?
- Discovering genetic variants linked to exercise‐induced atrial fibrillation in racehorses
- Exploring the molecular landscape of equine atrial fibrillation: A multiomics study
- The TASK‐1 potassium channel in equine atrial myocardium as a potential target to treat atrial fibrillation
- Preliminary validation of a smart textile device during high‐speed exercise for ECG quality & heart rate measurement
- Intra and interobserver reliability of pulsed‐wave tissue Doppler imaging of the left ventricle in healthy Standardbred neonatal foals
- Bicuspid pulmonary valve in horses: a case series
- Equid hepatitis B virus detected in two livers of donkeys with hyperlipemia
- Clinical presentation, treatment and outcome of foals with umbilical infections (2014‐2023)
- High prevalence of subclinical equid hepatitis B virus infection in a donkey farm in Romania
- Prevalence of *Strongylus vulgaris* in Danish horses and treatment response
- A retrospective study on intradermal allergy test (IDT) results and efficacy of allergen specific immunotherapy (ASIT) in horses with equine asthma (EA), equine allergic skin disease (EASD) and head shaking (HS)
- Prevalence of catheter‐associated thrombophlebitis in horses at a veterinary teaching hospital from 2012 to 2022
- Cystic calculi in horses: A retrospective study of 47 cases (2006‐2023)
- The role of oxidative stress in the equine asthma pathogenesis
- A retrospective study of the incidence and associated risk factors of exertional heat illness (EHI) in Thoroughbred racehorses in different states in Australia
- Retrospective study of congenital anomalies observed at necropsy from 2015 to 2023
- Selection of acute observation timepoints with intradermal skin testing in horses
- Are the clinical effects of mesenchymal stem cells merely mediated by phagocytosing macrophages?

## ORAL ABSTRACTS

**Oral Presentation**

Friday 15 November 2024, 11.30‐11.45

## Pharmacokinetics of co‐administration of misoprostol and sucralfate in adult horses

### **E.A. Sundman** ^1,2^, K. Gauthier^1^, P. Datta^3^, J. Joseph^4^, L.M. Hunyadi^1,2^

#### ^1^Texas Tech University School of Veterinary Medicine, Amarillo, USA; ^2^Advanced Equine Diagnostics, Kapaau, USA; ^3^Texas Tech University InfantRisk Center, Amarillo, USA; ^4^University of Tennessee College of Veterinary Medicine, Knoxville, USA

**Introduction**: Misoprostol and sucralfate are frequently utilized in the treatment of glandular gastric ulcers in horses. While sucralfate can bind with various medications, potentially affecting absorption when administered concurrently. However, co‐administration of oral medications is desirable in equine treatment protocols. The objective of the study was to evaluate the pharmacokinetic profile of misoprostol following a single dose of misoprostol alone in comparison to misoprostol co‐administrated with sucralfate in adult horses.

**Materials and Methods**: This study consisted of two‐phases, employing crossover pharmacokinetic study in eight horses. Treatment 1 included 5 mcg/kg misoprostol tablet (M). Treatment 2 involved the co‐administration of 5 mcg/kg misoprostol tablets with a 12 mcg/kg sucralfate tablet (MS). Both the treatments were administered as a top‐dress pellet. Plasma samples were collected for analysis of misoprostol concentrations using liquid chromatography–mass spectrometry. Standard single‐compartment pharmacokinetic parameters were calculated.

**Results**: Mean ± SD results were reported for C_max_ (pg/mL) (M: 96.4 ± 61.1; MS: 101.7 ± 77.3), T_max_ (h) (M: 0.8 ± 0.3; MS: 0.6 ± 0.2), T_1/2_ (h) (M: 0.9 ± 0.7; MS: 1.6 ± 1.5), mean residence time (h) (M: 1.1 ± 0.4; MS: 1.2 ± 0.4), and AUC_last_ (pg h/mL) (M: 113.5 ± 94.4; MS: 194.7 ± 195.9). No statistical difference was identified in misoprostol pharmacokinetic parameters when administered alone in comparison to co‐administration with sucralfate.

**Conclusion**: This study confirmed that sucralfate did not result in a statistically significant change in misoprostol absorption when co‐administered.

**Clinical Relevance**: Additional work is needed to evaluate the clinical effect of administration dosage regimens in horses with glandular gastric ulcers. Co‐administration of misoprostol with sucralfate may be a viable approach when treating horses clinically.

**Oral Presentation**

Friday 15 November 2024, 11.45‐12.00

## Comparing the inter‐ and intra‐rater reliability of two scoring systems for equine gastric glandular disease

### **C. Gilders** ^1^, L. Katz^1^, M. Hewetson^2^, V. Duggan^1^

#### ^1^University College Dublin, Dublin, Ireland; ^2^Royal Veterinary College, Hatfield, United Kingdom

**Introduction**: The grading of Equine Gastric Glandular Disease lesions remains a controversial topic. A previously recommended system of descriptive terminology has shown poor inter‐rater reliability. A modified 0 to 4 grading system has shown good inter‐ and intra‐rater reliability. This study was designed to compare the descriptive terminology system with the 0 to 4 grading system. The hypothesis was that there would be increased inter‐ and intra‐rater reliability with the 0 to 4 grading system.

**Methods**: Gastroscopy images of 30 glandular lesions of varying types and severity were assessed in duplicate under masked review by 3 board‐certified internal medicine specialists using both scoring systems. Images were viewed in a random order. Krippendorf's Alpha was used to determine intra‐ and inter‐rater reliability of both systems and of individual criteria within the descriptive system. A non‐parametric Kruskal‐Wallis test was used to evaluate correlations between numerical grades and descriptive terms; *P* ≤ .05 was considered significant.

**Results**: Numerical grade (*α* = 0.96), severity (*α* = 0.92), appearance (*α* = 0.91), topography (*α* = 0.92), and distribution (*α* = 0.88), each as sole descriptive criterion, showed near perfect intra‐rater agreements. Numerical grade (*α* = 0.66) and severity (*α* = 0.68), as sole descriptive criterion, also showed substantial inter‐rater agreements, although what was considered ‘normal’ epithelium varied between assessors. There was a significant association between numerical grade and severity (*P* < .0001). When all 4 descriptive terms were combined there was poor inter‐rater agreement (*α* = 0.07). Generally, erythematous lesions were given lower numerical grades (median = 1, ranging from 0 to 3) than any of the other descriptive terms (median = 3, ranging from 2 to 4). There were no significant associations between any descriptive term and numerical grade among assessors.

**Conclusion and Clinical Relevance**: Numerical grade and severity appear comparable; numerical grade showed better intra‐ and inter‐rater agreement than the descriptive system.

Limitations: use of still images and visualized on individual screens.

**Oral Presentation**

Friday 15 November 2024, 12.00‐12.15

## Pituitary gland abscessation after maxillary cheek tooth extraction in three horses

### **A.L. Hendrikx** ^1^, A.J. Brom‐Spierenburg^1^, E.W. Siegers^1^, C.M. Westermann^1^, W. Bergmann^1^, R.M.A.C. Houben^1^

#### ^1^Utrecht University, Utrecht, The Netherlands

**Introduction**: Reports of pituitary gland abscessation are rare in horses.

**Methods**: This case series describes three horses diagnosed with pituitary gland abscessation at the Equine Veterinary Teaching Hospital at Utrecht University, between 2005 and 2023.

**Results**: A (partial) extraction of a maxillary cheek tooth was performed in a 19‐year‐old Welsh pony mare (horse 1), a 6‐year‐old Standardbred gelding (horse 2) and a 16‐year‐old Trakehner gelding (horse 3), because of alveolitis of 207 (horse 1) and 209 (horse 2 and 3). The three horses developed pyrexia 1 to 11 days post‐extraction and showed signs of a central nervous system disorder including dullness, muscle tremors and ataxia. All horses were treated with antimicrobials, nonsteroidal anti‐inflammatory drugs and dexamethasone. Antimicrobial treatment included gentamycin and penicillin in all horses initially, in horse 1 followed by metronidazole and trimethoprim/sulfonamide. Horse 1 and 3 were additionally treated with vitamin B1, intravenous fluids and omeprazole. All three horses were euthanized due to marked deterioration of the neurological signs despite treatment. On post‐mortem examination all horses were found to have pituitary gland abscessation; additionally, a concurrent meningitis was found in all horses and an embolic pneumonia was found in two horses (horse 1 and 2). Horse 1 had a concurrent osteomyelitis in the bone surrounding the ventral aspect of the pituitary gland. Bacterial culture from the pituitary gland was performed only in horse 2 and 3 and *Bacteriodes* spp. was cultured from both horses. In horse 3, additionally a mixed culture with other anaerobic bacteria was found.

**Discussion/clinical relevance**: Pituitary gland abscessation should be considered as a differential diagnosis in horses with fever and signs of central nervous system disorder after maxillary cheek tooth extraction. Anaerobic bacteria including *Bacteriodes* spp. may be involved.

**Oral Presentation**

Friday 15 November 2024, 14.30‐14.45

## The seasonality of insulin concentrations in horses

### **A.L. Lopes** ^1^, A.D. Durham^1^, L.H. Huber^2^

#### ^1^Liphook Equine Hospital, Liphook, United Kingdom; ^2^Auburn University, Auburn, USA

**Introduction**: Measurement of serum insulin concentrations is the basis of diagnosis, monitoring, and prevention of insulin dysregulation and insulin‐associated laminitis. Current reference values do not consider seasonal variations in insulin concentrations and evidence available is limited. Our aim was to investigate the seasonality of insulin.

**Methods**: Analysis of laboratory data from the Liphook Equine Hospital from 2012 to 2023. Cases were included where submissions requested resting insulin. Other endocrine variables were also measured in many of the cases. Breed effect was assessed in the 9 most prevalent breeds: Arab, Cob, Connemara, Irish Sport Horse, New Forest, Shetland, Thoroughbred, Warmblood and Welsh; and donkey. Multivariate analysis was used to investigate a seasonal effect alongside other variables.

**Results**: In total, >50 000 submissions requested insulin analysis. In cases where it was requested, basal ACTH concentrations showed expected seasonal changes, as previously published. Insulin showed significant seasonal variability and was highest in winter (estimate = 2.2; SE ± 0.3; *P* < .001) and lowest in late summer and autumn (estimate = −0.4 and −0.7, respectively; SE ± 0.1; *P* < .001) compared with spring. The circannual pattern was broadly similar for all breeds except for resting insulin values in Thoroughbreds which had higher values in June and donkeys which had the highest insulin in May.

**Discussion**: Circannual changes in insulin showed higher values through winter and the lowest values through the summer and autumn. This could reflect a physiologic adaptation to increasing sugar ingestion through the grazing season being associated with a diminishing B‐cell response. Alternatively, seasonal changes in peripheral insulin sensitivity (therefore resting insulin concentrations) could lie behind this observation.

**Clinical relevance**: An improved understanding of insulin seasonality may improve the interpretation of laboratory data and management of clinical cases.

**Oral Presentation**

Friday 15 November 2024, 14.45‐15.00

## Retrospective case series on short‐term responses and lipoprotein profiles following treatment with dapagliflozin or ertugliflozin in horses with insulin dysregulation

### T.M. Sundra^1,2^, **E.J. Knowles** ^3,4^, D.I. Rendle ^5^, E. Kelty^6^, G.D. Lester^7^, G. Rossi^2^

#### ^1^Avon Ridge Equine Veterinary Services, Brigadoon, Australia; ^2^School of Veterinary Medicine, Murdoch University, Murdoch, Australia; ^3^Bell Equine Veterinary Clinic, Mereworh, United Kingdom; ^4^Royal Veterinary College, London, United Kingdom; ^5^EMT Consulting, Tiverton, United Kingdom; ^6^School of Population and Global Health, The University of Western Australia, Crawley, Australia; ^7^Equiimed, Perth, Australia

**Introduction**: Sodium‐glucose cotransporter 2 inhibitors (SGLT2i) are prescribed to manage hyperinsulinemia but the effects of dapagliflozin have not been investigated. Although hyperlipaemia is very rare, hypertriglyceridemia is commonly associated with SGLT2i treatment and investigation of the lipoprotein profiles is warranted.

**Methods**: Retrospective analysis of clinical records and stored serum from horses with hyperinsulinemia that received dapagliflozin (0.02 mg/kg, [n = 34]) or ertugliflozin (0.05 mg/kg [n = 24]) PO SID for 30 days. Within‐horse changes, correlations between variables, and differences between treatments were assessed using Wilcoxon signed‐rank, Spearman's rank correlation coefficient (rho) and the medians tests, respectively.

**Results**: Between day 0 (pre‐treatment) and day 30 within‐horse changes (median, inter‐quartile range [IQR]) were: basal serum [Insulin] (uU/mL) reduced from 170 (92‐280) to 28.7 (14.5‐90) (*P* < .0001), lameness grade (scale 0‐12) reduced from 6 (4‐10) to 2 (0‐2) (*P* < .001), serum [triglyceride] (mmol/L) increased from 0.5 (0.3‐0.6) to 1.0 (0.6‐1.56), [β‐hydroxybutyrate] (μmol/L) increased from 0.22 (0.17‐2.7) to 0.30 (0.24‐0.35) (*P* < .0001), [total cholesterol] (mmol/l) increased from 2.36 (2‐2.6) to 2.84 (2.4‐3.7) (*P* < .0001) and, as a percentage of serum lipids, high‐density lipoprotein (HDL) reduced from 52.4% (47.9%‐61.0%) to 50% (41%‐54.8%) (*P* = .034), very‐low density lipoprotein (VLDL) increased from 10.4% (6.4%‐14.4%) to 12.3% (9.9%‐16.8%) (*P* = .005). Differences between ertugliflozin and dapagliflozin groups in these parameters were not significant at day 0 or 30. At day 30, 10/48 (21%) cases had [triglycerides] >2.0 mmol/L (maximum = 10.8 mmol/L). Day 30 [triglyceride] was correlated with day 0: basal insulin (*P* < .001, rho = 0.47), [triglyceride] (*P* = .003, rho = 0.42) and %VLDL (*P* = .019, rho = 0.34) and day 30: [total cholesterol] (*P* < .001, rho = 0.67), %HDL (rho = −0.432, *P* = .014) and %VLDL (rho = 0.708, *P* < .001).

**Discussion and clinical relevance**: Dapagliflozin or ertugliflozin treatment is associated with reductions in [insulin] and lameness grade. Changes in [triglyceride] and lipoprotein profiles were usually minor with occasional marked hypertriglyceridemia. [β‐hydroxybutyrate] increased indicating ketosis, a metabolic pathway previously not thought to be relevant in horses.

**ORAL PRESENTATION ACVIM Resident Research Abstract Award Winner**

Friday 15 November 2024, 15.05‐15.20

## A high‐protein meal is associated with increased glucose‐dependent insulinotropic polypeptide secretion in insulin‐dysregulated horses

### Allison Palmer, DVM – The Ohio State University, Columbus, Ohio, USA

**Background**: The incretin hormones glucose‐dependent insulinotropic polypeptide (GIP) and glucagon‐like peptide 1 (GLP‐1) augment post‐prandial insulin secretion. Managing equine insulin dysregulation (ID) often involves feeding high‐protein ration balancers. Recent studies suggest that dietary amino acids can promote GIP secretion, enhancing post‐prandial [insulin]; this may increase the risk of hyperinsulinemia following consumption of high‐protein meals in horses with ID.

**Hypothesis**: Consumption of high‐protein meals will increase post‐prandial [GIP] and [GLP‐1] in horses with experimentally‐induced ID. Animals: Adult light‐breed horses with normal [ACTH] (n = 7).

**Methods**: Each horse underwent a frequently‐sampled insulin‐modified IV glucose tolerance test to characterize systemic insulin/glucose dynamics and a feed challenge test (FCT; 1 kg ration balancer [min 32% CP, max 13% NSC] consumed within 15 minutes, [GIP] and [GLP‐1] measured 0‐240 minutes afterward). Both tests were repeated after induction of ID (dexamethasone, 0.08 mg/kg PO SID, 7 days). Outcomes, including [GIP] and [GLP‐1] during the FCT, were compared between baseline and ID.

**Results**: [GIP] and [insulin] increased after a high‐protein meal; ID AUC‐GIP (1166 ± 363 pg/mL) was significantly higher than baseline AUC‐GIP (767 ± 199 pg/mL; *P* = .014). There was no difference in AUC‐GLP‐1 between baseline (50.7 ± 16.6 pg/mL) and ID (39.1 ± 25.3 pg/mL; *P* = .47).

**Conclusions and Clinical Importance**: Horses with experimentally‐induced ID displayed significantly greater GIP responses to a high‐protein meal than at baseline, suggesting that GIP plays a role exacerbating post‐prandial hyperinsulinemia in this context.

**Oral Presentation**

Friday 15 November 2024, 15.20‐15.35

## Preliminary results on the measurement of beta‐endorphins in horses with pituitary pars intermedia dysfunction

### **N.E. Fouché** ^1^, M. Farinha do Sul^1^, G. Christen^1^, R. Bruckmaier^2^, J. Gross^2^, C. Philipona^2^, N. Besuchet Schmutz^2^, J. Howard^3^, V. Gerber^1^

#### ^1^ISME Swiss Institute of Equine Medicine, Bern, Switzerland; ^2^Division of Veterinary Physiology, Bern, Switzerland; ^3^Clinical Diagnostic Laboratory, Bern, Switzerland

**Introduction**: Pituitary pars intermedia dysfunction (PPID) is a common disorder in aged horses, associated with overproduction of proopiomelanocortin‐derived peptides, including beta‐endorphins. Three reports from the early 1990s demonstrated elevated beta‐endorphin concentrations in horses with PPID compared with controls.

**Methods**: A competitive immunoassay, designed for measuring beta‐endorphin concentrations in human samples, was validated for use in equine serum. Beta‐endorphin concentrations were measured in July in horses with PPID (n = 17; ACTH: 76.4‐1251 pg/mL) and healthy controls (n = 17; ACTH 9.7‐27.4 pg/mL), and in both July and October in horses with PPID (n = 8; ACTH: July 51.9‐433.4 pg/mL, October 98.8‐1251 pg/mL) and healthy controls (n = 8; ACTH: July 5.6‐16.7 pg/mL, October 11.5‐42 pg/mL). Groups were defined based on ACTH concentrations.

**Results**: The within‐ and between‐assay CVs of the assay were 3.37% and 3.24%, respectively. Beta‐endorphin concentrations were significantly higher (*P* < .01) in horses with PPID (median, 195 pg/mL; range, 9‐1440 pg/mL) compared with controls (median, 22 pg/mL; range, 5‐64 pg/mL). ACTH and beta‐endorphin concentrations were significantly correlated in horses with PPID (*ρ* = 0.5; *P* = .04) but not in controls. Regarding seasonal differences, beta‐endorphin concentrations were significantly higher in horses with PPID in July (median, 165 pg/mL; range, 47‐950 pg/mL) and October (median, 506 pg/mL; range, 211‐1764 pg/mL) compared with controls (July: median, 74 pg/mL; range, 19‐167 pg/mL; October: median, 217 pg/mL; range, 33‐474 pg/mL). Beta‐endorphin concentrations measured in July that exceeded 91 pg/mL exhibited a sensitivity and specificity of 76% and 96%, respectively, for detecting PPID (AUC 0.90).

**Conclusions**: The assay used is suitable for the measurement of equine beta‐endorphin concentrations. Our analysis demonstrates that beta‐endorphins are higher in horses with PPID compared to controls and that concentrations surpassing 91 pg/mL show diagnostic potential for detecting PPID.

**Clinical relevance**: This study serves as a basis to investigate the role of beta‐endorphins in the pathophysiology and diagnosis of equine PPID.

**Oral Presentation**

Friday 15 November 2024, 15.35‐15.50

## Vena contracta and proximal isovelocity surface area as potential echocardiographic measurements for severity grading of mitral valve regurgitation

### **M. Demeyere** ^1^, A. Vogelsang^1^, A. Decloedt^1^, G. Loon^1^

#### ^1^Ghent University, Faculty of Veterinary Medicine, Merelbeke, Belgium

**Introduction**: Vena contracta (VC) and proximal isovelocity surface area (PISA) measurements are commonly used in human cardiology to assess mitral valve regurgitation (MR) severity. This preliminary retrospective study aimed to investigate whether VC and PISA measurements can be used to distinguish different grades of MR in horses.

**Methods**: VC and PISA measurements were performed on cardiac examinations of 80 horses with MR, aged 7 ± 6 years. The VC width and radius, flow, and area of the PISA were measured on a right and left parasternal view using color flow Doppler. From the right parasternal view MR could only be visualized in 53 horses. MR was graded according to the MR grading system used by the Equine CardioTeam Ghent as trivial, mild, moderate, or severe. Each group consisted of 20 horses. Since data were non‐normally distributed, the Kruskal‐Wallis test was used for pairwise comparisons.

**Results**: On a left parasternal view, VC width and PISA (radius, flow, and area) measurements (n = 80) showed a significant difference between the following groups of MR: trivial‐moderate (*P* < .001), trivial‐severe (*P* < .001), mild‐moderate (*P* < .03), mild‐severe (*P* < .001). However, all measurements displayed a considerable amount of variation, illustrated here with the VC data: trivial: 0.37 cm [0.10‐0.50], mild: 0.61 cm [0.20‐1.50], moderate: 0.99 cm [0.40‐1.60], severe: 1.47 cm [0.60‐2.30]. Right parasternal images (n = 53) only showed a significant difference between trivial‐severe (*P* = .038), mild‐moderate (*P* = .048), and mild‐severe (*P* = .008) in the radius and area PISA measurements. PISA flow only showed a significant difference between mild‐severe MR (*P* = .006).

**Conclusion**: VC and PISA (radius, flow, and area) measurements on a left parasternal view differed significantly between different MR grades and were superior to the same measurements on a right parasternal view.

**Clinical relevance**: Since VC and PISA measurements can be used to distinguish certain grades of MR, they may have an added value in improving MR diagnosis. However, measurement variability still needs to be determined.

**Oral Presentation**

Friday 15 November 2024, 11.30‐11.45

## Development and application of the EFFE (ecographic fast foal evaluation) protocol: A pilot study

### **N.E. Ellero** ^1^, A.M. Maggi^1^, I.I. Imposimato^1^, A.L. Lanci^1^, J.M. Mariella^1^, C.C. Castagnetti^1^, F.F. Freccero^1^

#### ^1^Department of Veterinary Medical Sciences, University of Bologna, Bologna, Italy

**Introduction**: Point‐of‐Care Ultrasound protocols in adult horses (eg, FLASH and CRASH) are increasingly used in emergency scenarios. This pilot study aimed to develop and apply a focused ultrasound (US) protocol to assess specific thoraco‐abdominal windows in foals.

**Methods**: EFFE (Ecographic Fast Foal Evaluation) protocol included 12 thoraco‐abdominal US windows from left and right recumbency (4 right‐side, 3 ventral, 5 left‐side) using a curved array probe (frequency 5‐1 MHz). To test feasibility and preliminary diagnostic performances, qualitative imaging score (QIS; 0‐3), 2D‐US findings, small intestine motility score (SIMS; 1‐4) and diagnosis were described in a cohort of healthy (n = 12) and sick (n = 14) foals. Images were blindly reviewed by the same observer.

**Results**: Acquisition time was relatively long but improved along with experience. In healthy and sick foals (QIS, 2.6 vs 2.3), stomach was visible in 100% vs 71% in a ventral view (cranio‐caudal diameter, 5.4 vs 6.2 cm; wall thickness, 0.40 vs 0.39 cm), duodenum in 92% vs 83% in a right‐side view (1 vs 0.78 contractile act/10 seconds; wall thickness, 0.25 vs 0.23 cm), small intestine in 100% in a ventral view (SIMS, 3.9 vs 3.2, *P* = .04; wall thickness, 0.23 vs 0.24 cm), large intestine (wall thickness, 0.33 vs 0.32 cm) and urinary bladder in 100% of cases in a right‐side and ventral view, respectively. EFFE imaging findings agreed with the clinicians' diagnosis in all sick foals with strangulating (eg, intussusception), non‐strangulating intestinal lesions (eg, enteritis), and peritoneal effusions (eg, uroperitoneum and septic peritonitis) (n = 7). Three/12 views were poorly performing.

**Discussion**: The overall feasibility of EFFE protocol was good. For optimization, left‐side views may not be performed, and total windows reduced to 4 right‐side and 3 ventral views in the left recumbency only.

**Clinical Relevance**: Once validated, EFFE protocol may become a useful tool in emergency settings for rapid US diagnosis of major abdominal equine neonatal conditions.

**Oral Presentation**

Friday 15 November 2024, 11.45‐12.00

## Neuronal and astroglial dysfunction in critically ill neonatal foals

### **K.D. Dembek** ^1^, J. Perez^1^, K. Coley^1^, D. Wong^2^, N. Browne^3^, E. Elder^4^

#### ^1^North Carolina State University, Raleigh, North Carolina, USA; ^2^Iowa State University, Ames, Iowa, USA; ^3^Hagyard Equine Medical Institute, Lexington, Kentucky, USA; ^4^Humphrey, Giacopuzzi & Associates, Somis, California, USA

**Introduction**: Neurological dysfunction is often recognized in critically ill foals presented for sepsis or neonatal maladjustment syndrome (NMS). Astrocyte and neuronal proteins such as glial‐fibrillary‐acidic protein (GFAP), astrocytic‐protein‐S100B, and brain‐derived neurotrophic factor (BDNF) may be used for monitoring and prognosis in critically ill foals. The goal of this study was to measure the plasma concentration of biomarkers of neurological damage in healthy and critically ill foals and to determine their association with outcome.

**Methods**: Biomarker concentrations were measured in foals (<7 days old) upon admission and at 24 and 48 hours of hospitalization in this prospective, longitudinal study. Based on clinical and laboratory findings foals were categorized into 4 groups: healthy (n = 8), septic (n = 17) with sepsis score >12, sick non‐septic [SNS] (n = 19), and NMS (n = 14). The plasma concentration of biomarkers was determined using ELISA and single‐molecule‐array technology, and the data were analyzed using parametric methods.

**Results**: Baseline BDNF concentration was higher compared to 24 and 48 hours in septic foals (*P* < .05). In NMS foals, BDNF concentration decreased over the first day of hospitalization and was lower compared to healthy foals at 24 hours (*P* < .05). Baseline GFAP concentration was decreased in all 3 groups of foals compared to healthy. NMS and SNS had lower concentrations of GFAP at 24 and 48 hours compared to healthy foals (*P* < .05). In SNS foals, S100B concentrations decreased from baseline over 24 hours (*P* < .05). Non‐survivors had a decreased baseline concentration of GFAP and increased concentration of BDNF compared to survivors (*P* < .05).

**Discussion and clinical relevance**: An increased concentration of BDNF on admission in septic and NMS foals is consistent with its role in postnatal brain development, synaptogenesis, and survival. Reduced GFAP concentration in NMS and septic foals suggests astroglial dysfunction or delayed postnatal astrogliogenesis. Both BDNF and GFAP may be used as prognosticating factors in critically ill foals.

**ORAL PRESENTATION ACVIM Resident Research Abstract Award Winner**

Friday 15 November 2024, 12.00‐12.15

## Biomarkers of brain injury in foals with neonatal maladjustment syndrome

### Javier Perez Quesada, DVM – North Carolina State University, Raleigh, North Carolina, USA

**Background**: Neonatal maladjustment syndrome (NMS) is a common disease of foals resulting in neurological dysfunction and increased mortality. Plasma biomarkers of brain injury, such as brain‐derived neurotrophic factor (BDNF), glial‐fibrillary‐acidic protein(GFAP), and astrocytic‐protein‐S100B may be used for diagnosis and monitoring of foals with NMS.

**Objectives**: To measure plasma concentration of biomarkers of neurological damage (BDNF, GFAP, and S100B) in foals with NMS, foals presented for other diseases (sick‐foals), and healthy foals, and determine their association with outcome. Animals: 8 healthy foals, 10 NMS foals, 16 sick‐foals hospitalized for other diseases (eg, diarrhea) < 7 days of age. Of the NMS and sick‐foals, 20 survived and 6 did not.

**Methods**: Biomarker concentrations were determined in all foals on admission and at 24, 48, and 72 hours of hospitalization in this prospective, longitudinal study. Plasma concentration of biomarkers was measured with ELISA and single‐molecule‐array technology. Data were analyzed with parametric methods.

**Results**: GFAP concentration was decreased in NMS and sick‐foals compared to healthy foals at time 0 (0.9 ± 0.52, 0.6 ± 0.21, 3.7 ± 1.2 ng/mL), 24 hours (0.84 ± 0.22, 0.73 ± 0.25, 3.6 ± 1.03), and 48 hours (0.5 ± 0.1, 0.56 ± 0.12, 3.1 ± 0.8) (*P* < .05), respectively. Non‐survivors had a decreased concentration of GFAP (0.4 ± 0.12 ng/mL) compared to healthy foals (3.7 ± 1.2) and survivors(1.2 ± 0.21) over the first 24 hours of hospitalization (*P* < .05). BDNF and S100B concentrations were not different between groups of foals or time points (*P* > .05).

**Conclusions**: Reduced GFAP concentration in NMS and sick‐foals suggests astroglial dysfunction or delayed postnatal astrogliogenesis. GFAP may be used as a prognosticating factor in critically ill foals.

**Oral Presentation**

Friday 15 November 2024, 14.30‐14.45

## Equine corona virus infections in adult horses: Investigating seroconversion and fecal shedding patterns

### **H.C. Smith** ^1^, M.J.P. Theelen^1^, R.M.A.C. Houben^1^, R. Boon^1^, L. Wollenberg^2^, C. Maanen^2^

#### ^1^Utrecht University, Faculty Veterinary Medicine, Utrecht, The Netherlands; ^2^Royal GD, Deventer, The Netherlands

**Introduction**: Equine Corona Virus (ECoV) causes outbreaks of anorexia, pyrexia, lethargy, and enteric disease in horses. In this study we aim to (1) evaluate if horses seroconvert after clinical ECoV infection and (2) to investigate the pattern of fecal excretion of ECoV to determine the optimal testing moment.

**Methods**: We performed a prospective observational study of a naturally occurring outbreak of ECoV including 16 horses presenting with clinical complaints. Serum was collected twice from each horse: on the first day of clinical signs (D1) and 16 days later (D16). Seroconversion was defined as a change from negative to positive and a significant increase as at least 0.6 sample to positive ratio units (S/P units) between acute and convalescent samples. Clinical data and fecal swabs for PCRs were collected on D1, D2, D3, D4, D7, D10, D13, and D16.

**Results**: Eight out of sixteen horses 50% (95% CI 26%‐75%) seroconverted or showed a significant increase in S/P units by D16 and 11/16 horses 69% (95% CI 46%‐91%) had a positive PCR result at least once within the study period. Shedding was intermittent, some horses tested negative before then testing positive again. Day 2 had the greatest number of positive PCR results; 6/16 horses 38% (95% CI 14%‐61%). At day 16, 3/16 horses 19% (95% CI 0%‐38%) were still shedding ECoV.

**Discussion**: Seroconversion or a significant increase in S/P units, identified 50% of cases with ECoV complaints. Day two after the start of clinical signs was the most likely day to detect ECoV by PCR in our study. Fecal shedding was found to be intermittent over the course of 16 days.

**Clinical relevance**: Submitting fecal samples over multiple days might improve chances of detecting ECoV by PCR. Demonstrating seroconversion or a significant increase in S/P units could be a useful adjunctive test to PCR.

**Oral Presentation**

Friday 15 November 2024, 14.45‐15.00

## Seroprevalence of West Nile, Usutu, and Tick‐borne encephalitis virus in equids from the southwest of France in 2023

### **N. Chevalier** ^1^, C. MIGNE^2^ , C. Bigeard^3^, M.H. Teheipuaura^2^, M. Dumarest^2^, M. Chevrier^2^, C. Marcillaud‐Pitel^4^, E. Querré^5^, M. Mas^5^, B. Durand^6^, C. Lupo^4^, A. Leblond^6^, M. Depecker^1^, G. Gonzales^2^

#### ^1^Clinique équine de conques, Saint aubin de branne, France; ^2^ANSES, Maisons alfort, France; ^3^ENSVI‐FVI, Marcy l'étoile, France; ^4^ Réseau d'Epidémio‐Surveillance en Pathologie Équine, Saint‐Contest, France; ^5^Ecole Nationale Vétérinaire de Toulouse, Toulouse, France; ^6^Epidémiologie des Maladies Animales et Zoonotiques, Marcy l'étoile, France

**Introduction**: In 2022, France faced the emergence of West Nile virus (WNV) on the Atlantic coast, in Gironde county. Three equine cases of WNV infection were diagnosed in October 2022 with symptoms consistent with ataxia, weakness, and/or encephalitis. We investigated the seroprevalence of WNV and the occurrence of orthoflaviviruses actively circulating (Usutu [USUV] and Tick‐borne encephalitis viruses [TBEV]) within three risk zones defined in Gironde containing wetlands and protected bird habitats.

The aim was to obtain an overview of orthoflaviviral circulation in Southwestern France and to evaluate risk factors associated with WNV seropositivity.

**Methods**: Serum samples were collected from 494 equids during spring 2023. They were first tested for IgG orthoflavivirus using a commercial pan‐flavivirus ELISA kit. Positive serum samples were then subjected to virus neutralization tests specific for WNV, USUV, and TBEV. Mixed‐effects logistic regression models were then performed.

**Results**: Our results confirm an active circulation of WNV in the Confluence area of Gironde (seroprevalence rate of 9%). The occurrence of USUV and TBEV in equids was estimated to 5% and 2%, respectively. For WNV, model selection allowed to identify two risk factors of VNT seropositivity in the Confluence zone: the type of housing with a strongly increased risk in animals always kept on pasture, and the distance to the nearest bird protection area, with a decreasing risk when this distance increased.

**Conclusions**: WNV seroprevalence rate estimated in horses in Gironde is close to that estimated on the Mediterranean basin. The type of housing and the distance to bird‐protected habitats were identified as risk factors associated with an increase of WNV seropositivity.

**Clinical relevance**: Active circulation of orthoflavivirus is reported for the first time in Gironde, reflecting epidemiological changes. Environmental factors and animal housing must be taken into account to mitigate the risk of exposure.

**Oral Presentation**

Friday 15 November 2024, 15.05‐15.20

## Comparison of the long‐term humoral immune response against West Nile virus in clinically and inapparently infected horses

### **C.H. Tolnai** ^1^, P.É. Forgách^1^, B. Paszerbovics^1^, O. Kutasi^1^

#### ^1^University of Veterinary Medicine Budapest, Budapest, Hungary

**Introduction**: West Nile virus (WNV) emerges into new territories and re‐emerges in endemic areas causing significant numbers of equine and human neurological cases annually. According to the literature, survivors develop life‐long immunity against the pathogen; however, the data are limited in horses. Our study aimed to compare the magnitude and length of humoral immune response in clinically and asymptomatically infected horses.

**Methods**: Serum samples were taken from naturally infected animals 1‐, 2‐ and 4 years post‐infection. A total of 19 clinically and 25 asymptomatically infected horses were enrolled into the study. The WNV‐neutralizing antibody (nAb) titers were measured. First, a WNV IgG ELISA was performed, followed by a micro‐wells virus neutralization assay.

**Results**: All clinically infected horses were WNV IgG positive during the study. Exactly 25/25 asymptomatically infected animals were seropositive in the first year of the study, afterwards, one horse became and remained seronegative. The mean nAb levels in the first, second, and fourth year of the study were 704 (SD = 554.6), 435.2 (SD = 352.8), and 554.6 (SD = 376.8) in clinically infected horses and 241.8 (SD = 334.5), 128.1 (SD = 134.9), and 15.1 (SD = 21.8) in asymptomatically infected animals, respectively. A significant difference (*P* = .0139) was found between the WNV nAb levels of clinically and asymptomatically infected animals. There was a significant decrease (*P* < .01) in titers over time among inapparently infected horses, while this was not statistically significant in clinically infected animals.

**Discussion**: The level of nAbs correlates best with the protection against Orthoflaviviruses. Clinically infected horses maintained protective WNV nAb levels 4 years post‐infection, however, the significantly decreased WNV titers of inapparently infected animals might not be protective against re‐infection.

**Clinical Relevance**: We recommend the regular monitoring of WNV nAb levels. Animals with inadequate protection should be vaccinated.

**Oral Presentation**

Friday 15 November 2024, 15.20‐15.35

## Sequencing bacterial cell‐free‐DNA to detect pathogenic bacteria in the blood of equine neonates; a pilot study

### **E.W. Siegers** ^1^, L.T. Chen^2^, A.E. Wesdorp^2^, M. Jager^2^, C. Vermeulen^2^, A.L. Zomer^1^, A.J. Brom‐Spierenburg^1^, C.M. Westermann^1^, E.M. Broens^1^, J.A. Wagenaar^1^, J. Ridder^2^, M.J.P. Theelen^1^

#### ^1^Utrecht University, Utrecht, The Netherlands; ^2^UMC Utrecht, Utrecht, The Netherlands

**Introduction**: Blood stream infections (BSI) are an important cause of death in neonatal foals. Current diagnostic methods are time‐consuming and lack sensitivity and specificity. In humans, bacterial cell‐free‐DNA (cfDNA) sequencing enables rapid detection of bacterial pathogens. The aim of this study was to explore the diagnostic potential of bacterial cfDNA sequencing in neonatal foals.

**Methods**: Eight healthy and 22 ill foals (<14 days) were included in this pilot study. Ill foals were classified based on neonatal systemic inflammatory response syndrome (nSIRS) scores: n = 10 positive (nSIRS+), n = 12 negative (nSIRS−). Blood samples were collected for cfDNA sequencing. Single‐stranded sub‐nucleosome‐size‐enriched DNA‐capture based library preparation was used to enrich for bacterial cfDNA, followed by untargeted Illumina sequencing. Total cfDNA yield, host mitochondrial cfDNA fraction and bacterial cfDNA fraction were compared between groups using a Kruskal‐Wallis and post‐hoc Dunn's test. The reads were decontaminated and classified to detect pathogenic bacterial species and compared to blood culture results.

**Results**: Bacterial cfDNA was detected in all foals at varying levels. No significant differences were observed in total cfDNA or bacterial cfDNA fraction between groups. Mitochondrial cfDNA fraction was lower in nSIRS+ compared to healthy foals (*P* = .013). In the nSIRS+ foals, 80% showed elevated bacterial counts compared to healthy background counts, whereas only 30% had positive blood cultures. Elevated numbers of bacterial genera associated with BSI were observed in the SIRS+ group compared to the SIRS− and the healthy group. The observed mapped bacterial species identified multiple species per individual foal.

**Discussion/clinical relevance**: in SIRS+ foals, 80% had elevated mapped bacterial cfDNA levels with multiple bacterial species identified, whereas only 30% of SIRS+ foals had a positive blood culture. Bacterial cfDNA sequencing holds potential as a new and potentially more sensitive diagnostic tool for detecting BSI in foals. These findings support further investigation to validate the technique.

**Oral Presentation**

Friday 15 November 2024, 15.35‐15.50

## Nosocomial syndrome surveillance in an equine veterinary teaching hospital: Prevalence and feasibility

### L. Liot^1^, **M. Allano** ^1^, T. Juette^1^

#### ^1^Université de Montréal, Saint‐Hyacinthe, Canada

**Introduction**: Healthcare‐acquired infections, or nosocomial syndromes (NS), are a health issue, requiring surveillance. There is little data in veterinary medicine. We aimed to estimate the prevalence of seven nosocomial syndromes in our teaching hospital and to study the feasibility of an active surveillance tool.

**Methods**: A paper‐based questionnaire was developed and pre‐tested. For each patient hospitalized at least one night for 8 weeks, data (signalment, occurrence of one or more SNs, procedures, and treatments) were collected prospectively and verified with medical records. A survey was submitted to respondents at the end of the collection period. Descriptive analyses were used, including prevalence estimations. The statistical effect of variables on prevalence was tested using a generalized linear mixed model (R software).

**Results**: Out of 116 equids, 29 (25%, 95% CI: 17‐32) presented at least one SN: mainly intravenous catheter site infections (11%), digestive syndromes (9%), fever of unknown origin (7%), surgical site infections (3%) and respiratory syndromes (2%). Sepsis and urinary inflammation were not detected. Six patients presented more than one SN. The variables gender, age, breed, duration of hospitalization, service of admission, and type of affection did not significantly affect the occurrence of SNs (*P* > .05). Respondents were satisfied with the tool but suggested some modifications.

**Conclusions and clinical relevance**: The prevalence of nosocomial syndromes was measured prospectively for the first time at our equine hospital, using an active surveillance tool. It appears applicable in the long term, even if adjustments will be necessary to improve data collection.

**Oral Presentation**

Saturday 16 November 2024, 10.45‐11.00

## Identifying the origin of left atrial ectopy, including pulmonary veins, via multiple catheter recording in the right heart

### **E. Buschmann** ^1^, G. Van Steenkiste^1^, I. Vernemmen^1^, M. Demeyere^1^, S. Schauvliege^1^, A. Decloedt^1^, G. Van Loon^1^

#### ^1^Ghent University, Merelbeke, Belgium

**Introduction**: Knowing the anatomical origin of atrial arrhythmias is essential before planning ablation. In humans and dogs, multiple catheter mapping allows to characterize arrhythmias but requires fluoroscopy, a poorly applicable technique in horses. The aim was to perform ultrasound‐guided multiple catheter mapping via the right heart during left atrial pacing in order to identify specific activation patterns characteristic for the origin of ectopy.

**Methods**: In seven anesthetized horses, under ultrasound guidance and confirmation by 3D mapping, four decapolar catheters were positioned at the crista terminalis, tuberculum intervenosum, caudal vena cava and coronary sinus (CS) for electrogram recording. After performing a transseptal puncture, a fifth catheter was used to pace (45/min) in the left atrium at left atrial appendage, septum, ostium I, II, III, and IV. Atrial activation timings were recorded for each pacing site, relative to the pacing stimulus (0 ms).

**Results**: Pacing the left atrial appendage sequentially activated CS (38[IQR 54] ms), tuberculum (100[32] ms), crista (115[43] ms) and caudal vena cava (127[53] ms). Pacing the septum activated the caudal vena cava (67[24] ms), tuberculum (71[22] ms), CS (87[24] ms), and crista (102[36] ms). Activation patterns from ostium I and II were: CS (60[20] ms; 76[24] ms, respectively), caudal vena cava (103[17] ms; 84[40] ms), tuberculum (124[34] ms; 119[92] ms) and crista (145[14]; 131[58] ms). Pacing ostium III activated tuberculum (66[18] ms), caudal vena cava (70[33] ms), crista (93[21] ms), and CS (123[13] ms). Pacing ostium IV activated CS (81[36] ms), tuberculum (107[44] ms), caudal vena cava (119[36] ms), and crista (139[16] ms).

**Conclusion**: Pacing‐induced ectopic depolarizations from the left atrium result in characteristic multiple catheter activation patterns.

**Clinical relevance**: Ablation of pulmonary veins has been performed experimentally but is very challenging, requiring a long anesthesia. Knowing the anatomical origin of atrial premature depolarizations is helpful to target specific pulmonary veins for ablation in order to reduce atrial fibrillation recurrence and might reveal which pulmonary veins are most often arrhythmogenic.

**Oral Presentation**

Saturday 16 November 2024, 12.00‐12.15

## Metformin mitigates atrial remodeling in horses with induced chronic atrial fibrillation

### **S.D. Nissen** ^1^, S.L. Haugaard^1^, M. Schneider^1^, S.A.T. Kjeldsen^1^, S.M. Sattler^1^, J.A. Bastrup^1^, J.B. Birk^1^, C. Hansen^1^, H. Carstensen^1^, C. Hopster‐Iversen^1^, A. Altintas^1^, T.A. Jepps^1^, S. Larsen^1^, R. Kjøbsted^1^, J.F.P. Wojtaszewski^1^, A. Saljic^1^, T. Jespersen^1^, R. Buhl^1^

#### ^1^University of Copenhagen, Copenhagen, Denmark

**Introduction**: Atrial fibrillation (AF) is a prevalent cardiac arrhythmia characterized by progressive atrial remodeling, challenging conventional therapy approaches. This study investigates the potential of metformin in preventing cardiac remodeling during chronic AF in horses. Metformin is an indirect AMP‐activated protein kinase (AMPK) activator and is currently used for treating equine metabolic syndrome, however, studies suggest that the activation of AMPK may be protective against AF.

**Methods**: Twenty retired Standardbred racehorses underwent continuous right atrial (RA) tachypacing until self‐sustained AF was achieved. Another four horses served as sham‐operated controls. Ten horses were treated with 30 mg/kg metformin orally twice daily during the study period and 10 served as controls. After 4 months of AF, AF inducibility and atrial refractoriness were assessed. The transcriptomic and proteomic changes of the posterior wall of the atria were assessed, followed by a comprehensive metabolic and structural tissue characterization.

**Results**: Metformin‐treated horses required significantly longer tachypacing compared to control horses (*P* = .02) and had longer RA effective refractory periods after 4 months of AF compared to controls (*P* = .03). Prolonged atrial refractoriness is considered to be protective against AF. RNA sequencing and proteomic analysis revealed upregulation of AMPK signaling pathways and mitochondrial function in metformin‐treated horses. Right atrial AMPKa2 activity was higher in metformin‐treated horses. However, metformin did not significantly alter mitochondrial ultrastructure or function. Our molecular analysis identified significant changes in protein levels regulating the atrial extracellular matrix, however, overall atrial collagen levels on histology did not differ between groups.

**Conclusion**: Four months of AF induced atrial electrical and molecular remodeling, but metformin treatment appeared to protect against AF initiation and progression.

**Clinical relevance**: Our findings support the use of metformin for AF prevention in horses and propose AMPK activation as a promising drug target for AF management.

**Oral Presentation**

Saturday 16 November 2024, 12.15‐12.30

## Heart rate variability based upon P‐wave indices in horses: An exploratory study

### **G. Van Steenkiste** ^1^, E. Van Den Branden^1^, A. Decloedt^1^, G. Van Loon^1^

#### ^1^Ghent University, Merelbeke, Belgium

**Introduction**: Heart rate variability (HRV) aims to assess autonomic tone by measuring variation in sinus node depolarization, usually measured by RR‐intervals. In horses, significant PQ variations and 2° atrioventricular‐blocks may affect results. Therefore, our study compared P‐wave and R‐wave intervals for HRV assessment in horses.

**Methodology**: Exactly 24‐hour resting ECGs were recorded from 9 healthy mares, clinically assessed to ensure absence of distress. ECG data were processed with custom Matlab scripts for filtering, normalization, and QRS‐complex detection. Automatic QRS‐markers were manually corrected. Bifid P‐waves were identified within specific windows set by Q onset and T‐wave offset, considering amplitude, slope, morphology, and prominence to detect P onset (Pon), P1 peak, and P2 peak. HRV metrics such as SD (SDNN), root mean square (RMSSD), Triangular Index (TRI), low‐frequency (LF), and high‐frequency (HF) components, and LF/HF have been calculated from the different P‐wave indices and RR intervals over 30‐minute time windows. A general mixed model with post‐hoc Bonferroni correction compared the HRV measures with marker type (P or R wave) as a fixed effect.

**Results**: HRV metrics for P2P2 intervals showed significantly lower SDNN, RMSSD, and TRI (mean difference −24.5, −11.9, −1.7, respectively; *P* < .001) but higher HF and LF/HF (0.069 and 0.008, *P* < .001) compared with RR intervals. HF was higher for P1P1 (0.093, *P* < .001). P1P1 intervals exhibited decreased SDNN (−23.1, *P* < .001) and LF/HF (−0.124, *P* < .001). PonPon and RR metrics did not differ significantly. The 2° atrioventricular‐blocks and large PQ variations were absent in this study sample.

**Conclusions**: P‐wave interval analysis is feasible for HRV analysis in horses, offering a potential advantage over RR interval assessment in more accurately reflecting autonomic nervous system function. Further studies on horses with more 2° atrioventricular‐blocks and PQ variations are needed to confirm.

**Clinical relevance**: P‐wave‐based HRV metrics may improve autonomic tone monitoring precision by mitigating atrioventricular nodal conduction effects.

**Oral Presentation**

Saturday 16 November 2024, 14.15‐14.30

## Effect of steamed hay on airway inflammation in horses with severe equine asthma

### **M. Leclère** ^1^, C. Raisky^1^, B. Mozo Vives^1^, L. Leduc^1^, A. Symoens^1^, T. Juette^1^, C. Bédard^1^

#### ^1^Université de Montréal, St‐Hyacinthe, Canada

**Introduction**: Although steaming hay reduces respirable particles, it has shown inconsistent results in clinical studies. The objective was to compare airway inflammation in horses with severe equine asthma (SEA) fed dry hay and steamed hay for a month. We hypothesized that horses with SEA would develop airway inflammation when fed dry hay but not steamed hay.

**Methods**: Nine horses with SEA from a research facility were selected. Before the study, they were maintained in remission on a pelleted diet, indoors. They were then fed steamed and dry hay for 4 weeks, in a prospective, cross‐over study, with a 4‐week washout period on a pelleted diet. Bronchoalveolar lavage fluid (BALF) differential cell counts were analyzed before and after 4 weeks of hay feeding. Data were analyzed using a linear mixed model with post‐hoc tests and Benjamini‐Hochberg corrections for multiple comparisons, and a Pearson correlation test was performed between BALF neutrophils with dry and steamed hay.

**Results**: Pulmonary neutrophils increased after the 4‐week period (time effect and post‐hoc End versus Baseline: *P* < .001), with no significant difference between dry and steamed hay (Baseline dry: mean 6.7% [SD 5.4]; End dry: 13.1% [6.0]; Baseline steamed: 5.6% [2.6]; End steamed: 10.5% [4.4]). There was no significant correlation between airway neutrophilia of horses fed dry hay and steamed hay (*r*: −.2, *P* = .7).

**Discussion**: Since all horses were housed in the same barn, those receiving steamed hay were also exposed to the dust generated by handling dry hay during feeding time. This nevertheless reflects common practice in many barns. The results are consistent with those of a previous study in which a short exposure (5 days) to steamed hay led to an increase in BALF neutrophils.

**Clinical Relevance**: Feeding steamed hay does not prevent airway inflammation in horses with severe asthma.

**Oral Presentation**

Saturday 16 November 2024, 14.30‐14.45

## Dust exposure and pulmonary inflammation in mild asthmatic horses fed pelleted or round bale hay: A pilot study

### **S. Preez** ^1^, S. Franklin^1^, J. Watson^1^, S. Gaskin^1^, Y.M. Tefera^1^, R. M.. Jurkowski^1^

#### ^1^The University of Adelaide, Adelaide, South Australia, Australia

**Introduction**: The mainstay of treatment and prevention of equine asthma (EA) is centered around reduction of organic dust exposure. Most of the organic dust originates from feed matter, rather than the environment.

**Objectives**: To evaluate respirable particle exposure and airway inflammation of horses fed a pelleted diet compared with round bale hay, using two feeder types.

**Methods**: Four horses, with mild EA (mEA) were housed in paddocks (40 m × 20 m) during a 16‐week cross‐over design, with 4‐by‐4‐week exposure periods. During exposure period one and three, all horses were fed a pelleted diet. During exposure period two and four, horses were in two groups (n = 2), each group was fed round bale hay from either a small‐hole‐net or metal feeder. Breathing zone dust particle collections and clinical examinations, including “improved clinically detectable equine asthma scoring system” (IDEASS) clinical score, were performed weekly and respiratory endoscopies, tracheal wash and bronchoalveolar lavage (BAL) were conducted fortnightly.

**Results**: Median (Inter Quartile Range) of respirable dust exposure was significantly higher in horses fed round bale hay, 0.180 (0.05‐0.41) mg/m^3^, in comparison with pelleted diet, 0.085 (0.04‐0.18) mg/m^3^, *P* = .037, whilst no significant differences were found between the net and metal feeder. The round bale diet resulted in significantly higher BAL neutrophil proportions compared to the pelleted diet, 4.67% (3.00‐8.08%) and 1.33% (1.00‐2.68%), *P* < .0001, respectively, with no significant difference between the net and metal feeder. Clinical score (mean rank) was significantly higher for the metal feeder (1.88) than the pelleted diet (1.13, *P* = .034), with no significant difference between the pelleted and net, nor the net and metal feeder diets.

**Discussion**: The complete pelleted diet resulted in lower respirable dust exposure, less pulmonary inflammation, and lower clinical score than the round bale diet.

**Clinical relevance**: Feeding round bale hay has a detrimental effect on the respiratory health of horses with mEA.

**ORAL PRESENTATION ACVIM Resident Research Abstract Award Winner**

Saturday 16 November 2024, 09.50‐10.05

## Characterization of renal lipidosis in equids: A postmortem case‐control study (2008‐2022)

### Kali Slavik, DVM – University of Pennsylvania, Philadelphia, Pennsylvania, USA

**Background**: Although reported in other species, findings associated with renal lipidosis have not been previously described in horses and donkeys.

**Objective**: To describe the signalment, clinicopathologic indices, and postmortem findings of equids with histologic diagnosis of both hepatic lipidosis and renal lipidosis (HL + RL) and compare to matched cases with hepatic lipidosis only (HL).

Animals: Twenty five equids with findings of renal and/or hepatic lipidosis at necropsy from a state diagnostic laboratory between 2008 and 2022.

**Methods**: Retrospective case‐control study. Signalment, history, affected system, and selected biochemical data were extracted from medical records. Each case of HL + RL was assigned a matched control from group HL for comparison of selected clinical data.

**Results**: Renal lipidosis was diagnosed in 0.55% of equid necropsies. Donkeys with hepatic lipidosis were more likely to also have renal lipidosis (7/13, 54%) compared to horses and ponies (18/197, 9%; *P* < .005). No cases of renal lipidosis were identified without concurrent hepatic lipidosis. Renal lipidosis cases most commonly presented with gastrointestinal (61%, 16/25) or neurologic (46%, 12/25) disease. Group HL + RL had a higher median intake plasma lactate (+6.2 mmol/L, IQR 0.4‐10.5; *P* = .04) and higher GGT activity (+246 U/L, IQR 72‐360; *P* = .02) compared to group HL controls. No significant differences between groups were noted in creatinine or triglyceride concentration.

**Conclusions**: Renal lipidosis is an occasional postmortem finding in equids with hepatic lipidosis, but is markedly more common in donkeys. Cases with renal lipidosis were not more likely to be azotemic than those with only hepatic lipidosis. The clinical significance of renal lipidosis is unknown.

**Oral Presentation**

Saturday 16 November 2024, 10.45‐11.00

## Exploring paraoxonase‐1 as a marker of inflammation and oxidative stress in horses with colitis

### **M. Winther** ^1^, J. Johnsson^2^, P. Madsen^2^, S. Jacobsen ^1^, T. Pihl^1^, S. Paltrinieri^3^, D. Scavone^3^, J. Céron^4^, L. Pardo‐Marin^4^

#### ^1^University of Copenhagen, Department of Veterinary Clinical Sciences, Copenhagen, Taastrup, Denmark; ^2^The Large Animal Teaching Hospital, Copenhagen, Denmark; ^3^University of Milan, Department of Veterinary Medicine and Animal Sciences, Milan, Italy; ^4^University of Murcia, Department of Animal Medicine and Surgery, Murcia, Spain

**Introduction**: Oxidative stress occurs due to imbalance between the production of reactive oxygen species and available antioxidants, and it is connected to inflammation. While the inflammatory response during equine colitis is well characterized, the role of oxidative stress is less understood. The novel marker paraoxonase‐1 (PON‐1) decreases during inflammation and oxidative stress, and its serum activity was therefore investigated in horses with colitis to assess its diagnostic and prognostic capacity.

**Methods**: PON‐1 activity was measured at admission in 161 horses with colitis and 57 healthy horses. Follow‐up samples from 106 horses with colitis were available. Correlation analyses were used to compare PON‐1 activity with known inflammatory‐ and disease severity markers (serum amyloid A [SAA], serum fibrinogen, serum iron, white blood cell count [WBC], heart rate [HR], blood lactate, packed cell volume [PCV]).

**Results**: Serum PON‐1 activity was significantly lower in horses with colitis (median [min‐max range] U/mL) (44.7 [3.8‐147.0]) compared to healthy horses (77.5 [11.3‐112.6]), and in non‐survivors (38.6 [3.8‐147.0]) compared to survivors (49.4 [8.8‐118.0]). However, PON‐1 activity could not reliably categorize horses as survivors/non‐survivors and the sensitivity and specificity were poor‐to‐modest. Activity did not change consistently over time in response to treatment. Serum PON‐1 activity was significantly but weakly correlated with SAA, HR, and blood lactate.

**Conclusion**: Despite statistical differences between groups, PON‐1 activity varied greatly within horses with colitis and there was no consistent change in activity, neither in horses with resolution of clinical signs (survivors) nor in horses that did not respond to treatment (non‐survivors). PON‐1 activity correlated only weakly with known inflammatory markers and disease severity markers and does not appear to reflect the clinical status of horses with colitis.

**Clinical relevance**: PON‐1 seems to add little to existing markers as it had suboptimal diagnostic performance and little prognostic abilities in horses with colitis.

**Oral Presentation**

Saturday 16 November 2024, 12.00‐12.15

## Evaluation of biomarkers (BIOs) in healthy and colic horses: correlation with systemic inflammatory response syndrome (SIRS) status and outcome

### **F. Bindi** ^1^, A. Elias‐Cortajarena^2^, L. De Marchi^1^, V. Vitale^2^, A. Spadari^3^, R. Rinnovati^3^, G. Sala^1^, F. Bonelli^1^, M. Sgorbini^1^

#### ^1^Department of Veterinary Sciences, University of Pisa, Pisa, Italy; ^2^Faculty of Veterinary Medicine, University CEU Cardenal Herrera, Valencia, Spain; ^3^Department of Veterinary Medical Sciences, University of Bologna, Bologna, Italy

**Introduction**: In colic horses, ischemic damage and inflammation have been attributed to morbidity and mortality and disease‐associated molecular changes. This study aimed to measure BIOs in healthy and colic horses to assess the correlation with SIRS status and outcome.

**Methods**: This prospective, multicentric study enrolled 78 horses: 10/78 were healthy, 68/78 were colic. At admission, 24, 48, 72, and 96 hours post‐hospitalization, colic horses underwent a complete physical exam, blood collection, and SIRS status assessment. A SIRS‐positive status was defined by the alteration of 2 or more of the following parameters: HR (>52 bpm), R > (>20 bpm), temperature (<37 or >38.5°C), WBC (<5 or >12.5 × 10^9^/L). Blood collection and SIRS status assessment were performed once in healthy horses. Data distribution was assessed using the Shapiro‐Wilk test. Differences in BIOs concentration among groups (SIRS‐positive, SIRS‐negative, survivor, and non‐survivor, accounting for sampling times), were analyzed using the Generalized Linear Mixed Models (IBM SPSS, BM‐Corporation, USA).

**Results**: Overall, 44/78 were SIRS‐negative and 34/78 SIRS‐positive horses. Significant differences were found between SIRS‐positive and SIRS‐negative horses for lipid peroxidation (*P* = .005), butyrylcholinesterase (*P* = .002), glutathione S‐transferase (*P* = .028), and glutathione peroxidase activities (*P* = .011). Significant differences were found between survivor and non‐survivor horses for lactate (*P* < .001), superoxide dismutase (*P* = .003), and total antioxidant capacity (*P* = .027).

**Discussion**: Colic horses exhibited BIOs' variations and alterations in relation to SIRS status and outcome. To date, studies have assessed these BIOs individually or in combination across different species and under various pathology, yielding heterogeneous results. To our knowledge, this is the first study assessing this panel of BIOs in relation to the SIRS status and outcome in colic‐afflicted horses.

**Clinical relevance**: The BIOs assessed in this study might be potential tools in determining the SIRS status and forecasting outcomes in colic horses. Further research is needed to clarify their potential clinical application in colic‐afflicted horses.

**Oral Presentation**

Saturday 16 November 2024, 12.15‐12.30

## Measurement of thymidine kinase‐1 activity in serum and body cavity fluids in horses with lymphoma, inflammatory bowel disease and other non‐inflammatory gastrointestinal disorders

### **K.D. Drozdzewska** ^1^, S.B. Burde^2^, E.M. Müller^2^, R.M. Merle^3^, H.G. Gehlen^1^

#### ^1^Equine Clinic, Freie Universitaet Berlin, Berlin, Germany; ^2^Laboklin Laboratory for Clinical Diagnostics GmbH & Co. KG, Bad Kissingen, Germany; ^3^Institute for Veterinary Epidemiology and Biostatistics, FU Berlin, Berlin, Germany

**Introduction**: Serum thymidine kinase‐1 activity (TK1_S_) is an internal tumor marker with variable accuracy. Measurement of TK1 in peritoneal/pleural fluid (TK1_PF_) remains unestablished in horses. The aim of this study was to compare both results.

**Methods**: TK1_S_ and TK1_PF_ were measured (chemiluminescent immunoassay) in horses with lymphoma, inflammatory bowel disease (IBD) and other non‐inflammatory gastrointestinal disorders (control).

Wilcoxon test compared TK1_S_ and TK1_PF_, while Spearman's rho (*ρ*) assessed correlation. Kruskal‐Wallis test examined group differences. Data were presented as median and interquartile range. Significance was set at *P* < .05.

**Results**: Among the 29 horses, 10 had lymphoma, 8 had IBD and 11 were controls. TK1_PF_ (3.52 U/L; 1.18‐14.15 U/L) was significantly higher (*P* = .001) than TK1_S_ (0.69 U/L; 0.49‐1.89 U/L). A moderate correlation between TK1_S_ and TK1_PF_ was observed (*ρ* = 0.42, *P* = .03), with the strongest correlation in control group (*ρ* = 0.77, *P* = .006). Significant differences were found in TK1_S_ between groups (*P* = .02), but not in TK1_PF_ (*P* = .35). TK1_S_ in horses with lymphoma (3.45 U/L; 0.65‐8.16 U/L) was significantly higher (*P* = .02) than in controls (0.49 U/L; 0.49‐0.75 U/L), but not significantly higher than in IBD (0.67 U/L; 0.49‐1.52 U/L).

**Discussion**: TK1_PF_ measurement proved feasible and can be performed on peritoneal/pleural fluid routinely taken during oncologic work‐up. Notably, TK1_PF_ showed a relevant rise compared to TK1_S_ in most horses with lymphoma, but elevation was observed in few horses with IBD as well. The main limitation was the small sample size, potentially preventing the detection of group differences. Additionally, inflammation accompanying gastrointestinal diseases might elevate TK1_S_ and potentially TK1_PF_.

**Clinical relevance**: Reference ranges for TK1_PF_ in horses should be evaluated. Further studies are needed to assess the usefulness of TK1_PF_ in lymphoma diagnostics and differentiation from other proliferative disorders, including inflammation.

**Oral Presentation**

Saturday 16 November 2024, 14.15‐14.30

## Histological and immunological comparison of gastrointestinal biopsies with their respective full‐thickness tissue counterpart in horses

### **D.J. Jean** ^1^, C.R. Ruault^1^, J.M. Monbrun^1^, T.J. Juette^1^, N.W. Wenzlow^2^

#### ^1^University of Montreal, St‐Hyacinthe, Canada; ^2^Texas Tech University, Amarillo, Texas, USA

**Introduction**: The histologic interpretation of gastrointestinal biopsies remains a challenge in horses, with lacking standards for these procedures.

**Objectives**: The study aimed to (1) describe and compared the histological and immunological findings in biopsy and full‐thickness gastrointestinal (GI) segments in horses.

**Methods**: Ten donated horses (6 females, 3 geldings and one stallion; 5‐28 years old) to the University of Montreal, without digestive conditions, were used. Full‐thickness and endoscopic forceps obtained mucosal biopsies were taken postmortem from the duodenum, jejunum and rectum in each horse. Standard hematoxylin eosin phloxine saffron (HEPS) sections from all GI tissues were evaluated blinded histologically by a board‐certified veterinary pathologist. Immunohistochemically stained slides allowed histomorphometrical counts (absolute numbers per field) of B lymphocytes (CD20) and T lymphocytes (CD3) within the duodenal and rectal epithelium, lamina propria (apical and basal areas) and the subcryptal areas (rectum only). CD3 and CD20 staining of biopsy samples were compared with the same immunohistochemical staining of three sections of their corresponding full‐thickness duodenal and rectal counterparts of equivalent size. Each section of full‐thickness tissue had similar size to the biopsies taken. Results were analyzed using intra‐class correlation (ICC).

**Results**: Preliminary data show that biopsies tissues tend to under‐estimate the number of lymphocytes and plasma cells in the duodenal lamina propria compared to their full‐thickness counterparts. Eosinophils are more prevalent in the jejunum compared to the duodenum. Rectal biopsies tend to have lower counts of neutrophils and macrophages compared to their full‐thickness counterparts.

**Discussion**: The trend to under‐estimate the immune cells in the duodenum biopsies compared to the full‐thickness duodenum and jejunum could hide a more severe and diffuse digestive infiltration.

**Clinical relevance**: These differences of immune cell distribution in the different sections of the horses' GI tract must be taken into consideration when interpreting GI tissues of equine patients.

**Oral Presentation**

Saturday 16 November 2024, 14.30‐14.45

## Genome‐wide association study introduced novel genomic loci of insect bite hypersensitivity in Finnhorses

### **M.J. Weckman** ^1^, N.P. Karikoski^1^, M.R. Raekallio^1^, L. Kvist^2^

#### ^1^University of Helsinki, Helsinki, Finland; ^2^University of Oulu, Oulu, Finland

**Introduction**: Equine insect bite hypersensitivity (IBH) is an allergic skin disease caused by the allergens in the saliva of *Culicoides* spp. The IgE‐mediated reactions are characterized with chronic seasonal pruritic dermatitis. Pruritis often results to severe skin lesions and significantly impairs the well‐being of horses. IBH is thought to be multifactorial; morbidity is influenced by both environmental and genetic factors.

**Methods**: DNA samples were collected from Finnhorses with typical, recurrent (at least 2 consecutive years) IBH symptoms (age minimum 3 years) and healthy controls (age minimum 10 years). The symptoms included pruritus, skin thickening, hair loss and scaling. A case‐control genome‐wide association study was performed using the Illumina's Equine 80K Genotyping BeadChip containing 65 157 single nucleotide polymorphisms (SNPs). PLINK v1.9 software were used for association analysis by applying the Fisher's exact test and for pruning and quality control.

**Results**: DNA samples were available from 72 cases (mean age ± SD 11.5 ± 5.9) and 72 controls (mean age ± SD 15.9 ± 4.7). The lowest *P*‐value was found in SNP BIEC2_1080912 (*P* = 9.241 × 10^−6^, OR = 3.719) which is in chromosome 9.

**Discussion and Clinical Relevance**: Protein coding gene close to the SNP with the lowest *P*‐value is a potential candidate gene for IBH. Candidate gene has role in the formation and regulation of several immune cells. Additionally, it promotes immune exhaustion. Further studies are required to clarify the role of this candidate gene in IBH in Finnhorses.

## POSTER ABSTRACTS

**Poster Presentation 1**

## Longitudinal changes in fecal short chain fatty acids during hospitalization in horses with different types of colic and their association with survival

### **C.L. Loublier** ^1^, M.C. Costa^2^, N.M. Moula^1^, C.D. Douny^1^, B.T. Taminiau^1^, G.D. Daube^1^, H.A. Amory^1^, C.C. Cesarini^1^

#### ^1^University of Liège, Liège, Belgium; ^2^Saint‐Hyacinthe University, Montreal, Canada

**Introduction**: Short chain fatty acids (SCFA) are end‐products of intestinal bacterial fermentation. Previous research has assessed longitudinal changes in the fecal bacterial microbiota of horses with colic, but evolution of their metabolome remains unknown. This study investigates SCFA dynamics in horses hospitalized with different colic types and their association with survival.

**Methods**: Twenty‐three horses with colic were prospectively studied, including 9 with inflammatory pathologies (INFL group), 9 with simple obstruction (SIMPLE group), and 5 with strangulated obstruction (STR group). Fecal SCFA (C2, C3, C4, iC4, C5, iC5, C6) were quantified by solid‐phase microextraction and gas chromatography‐mass spectrometry on days 1 (admission), 3, and 5 of hospitalization. Data were analyzed using a mixed model analysis including the effect of horse, group, and hospitalization duration, and considered significant when *P* < .05.

**Results**: Concentrations of C5 (*P* = .0346) and C6 (*P* = .0418) were significantly higher in INFL compared to SIMPLE group on admission. By day 3, C2, C3, and C4 were higher in SIMPLE compared to INFL group (respectively, *P* = .0035, .0218, and .0134), while iC4, iC5, C5, and total SCFA were higher in SIMPLE compared to INFL and STR group (respectively, *P* = .0015 and .0385; *P* = .0006 and .0185; *P* = .0061 and .0093; *P* = .0138 and .0335). On day 5, concentration of SCFA were not statistically different between groups. Survivors exhibited lower C2, C4, iC4, C5, iC5, and total SCFA on day 5 (respectively, *P* = .0057, .0296, .0044, .0443, .0102, and .0110).

**Conclusions**: Fecal SCFA profile in hospitalized horses with colic varies with disease type and survival status.

**Clinical relevance**: The use of fecal SCFA profile as outcome markers in horses with colic deserves further research.

**Poster Presentation 2**

## Fungal gastric lesions: Morphological, histopathological, and microbiological features in three horses

### **P. Neira Egea** ^1^, M. De la Cuesta Torrado^1^, C. Bautista Erler^1^, V. Vitale^1^, C. Cesarini^2^, C. Alamar Malvoisin^2^

#### ^1^Hospital Clinico Veterinario CEU, Valencia, Spain; ^2^Universidad de Liège, Liège, Belgium

**Introduction**: Fungal gastrointestinal infections are rare in the horse. *Candida* spp. are ubiquitous yeast, part of the skin and mucosal microbiota of human and animals, that can become opportunistic pathogens under suitable conditions. In the horse, gastrointestinal candidiasis has been described in neonatal foals but not in adults. In humans, *Candida* colonization has been associated to inflammatory bowel disease (IBD) and gastric cancer.

**Methods**: Our aim is to describe the morphological, histopathological and microbiological features of gastric lesions associated with fungal infiltration in 3 horses referred to tertiary hospitals for weight loss and other unspecific signs. Multiple ancillary test to investigate weight loss were performed. Common features, including results of gastroscopy, gastric and duodenal biopsies, fungal and bacterial culture of the gastric lesions will be described.

**Results**: Horses included a 3 year old Miniature stallion, a 10 year old KWPN gelding and a 11 year old crossbred mare. Grossly, fungal lesions appeared as irregular, multifocal, yellow/white pseudo‐membranous plaque‐like areas on the squamous gastric mucosa. Concomitant grade 2 ESGD was found in 2 horses and pyloric lesions in 1 horse. On histological examination, fungal involvement was confirmed in all cases by the presence of fungal hyphae infiltrating the superficial layers of the squamous mucosa. Fungal culture yielded *Candida* spp. in 2 horses and was negative in one. Bacterial colonization of gastric lesions was supported in 2 horses by histology or culture. Additionally, mild to moderate lymphoplasmacytic duodenitis was identified in all horses.

**Conclusion**: *Candida* spp. can be found in the stomach of adult horses associated to lesions with a characteristic yellow/white pseudo‐membranous aspect. As described in humans, concomitant IBD can be found in affected patients.

**Clinical relevance**: *Candida* spp. infection of the stomach can occur in adult equine patients. Further studies are needed to establish its clinical significance and a possible relationship with IBD.

**Poster Presentation 3**

## Clinical, clinicopathological, pathological and genetic findings in six franches‐montagnes foals with suspected hypertriglyceridemia‐induced pancreatitis

### **M. Wyler** ^1^, M. Drögemüller^2^, V. Gerber^1^, C. Gurtner^3^, S. Meister^3^, T. Leeb^2^, N.E. Fouché^1^

#### ^1^Swiss Institute of Equine Medicine (ISME), Bern, Switzerland; ^2^Institute of Genetics, Bern, Switzerland; ^3^Institute of Animal Pathology, Bern, Switzerland

**Introduction**: Severe acute pancreatitis associated with hypertriglyceridemia is rarely reported in foals. A genetic cause for suspected hypertriglyceridemia‐induced pancreatitis in foals has not yet been described.

**Methods**: The current abstract delineates a retrospective case series of six Franches‐Montagnes foals with confirmed pancreatitis at necropsy. Genetic analyses were carried out to identify the cause of the suspected hypertriglyceridemia‐induced pancreatitis.

**Results**: Median age of the affected foals at admission was 5 days (range 4‐93) and all six were fillies. The affected foals were inbred to a single stallion and their pedigrees were consistent with an autosomal recessive disease. Most common clinical signs included apathy, reluctance to nurse, fever, abdominal distension, and diarrhea. One foal primarily showed neurological signs attributed to hepatoencephalic syndrome. All foals displayed severe hypertriglyceridemia (mean 33.3 mmol/L ± SD 8.2; RI 0.16‐0.73 mmol/L). Lipase activity was measured in 4/6 cases with a median of 146 U/L (range 95‐648: RI 9‐21 U/L). All the foals were euthanized, and severe necrotizing pancreatitis was diagnosed at necropsy. A candidate causative genetic variant affecting the *LMF1* gene encoding lipase maturation factor 1 was identified. It was a frameshift variant, XM_023616679.1:c.369_373delinsTCT or XP_023472447.1:p.(Leu125Argfs*193). All six foals carried the mutant allele in a homozygous state.

**Conclusions**: A causal relationship between severe hypertriglyceridemia and the postmortem diagnosis of pancreatitis in the present cases is strongly suspected. A candidate variant in the *LMF1* gene for hypertriglyceridemia has been identified.

**Clinical relevance**: Pancreatitis should be considered in the differential diagnosis of sick foals with diarrhea, colic, and possibly neurologic signs, especially those with high triglyceride levels. Since a genetic predisposition seems to be associated with hypertriglyceridemia and pancreatitis, genetic testing and strict avoidance of carrier × carrier matings should be implemented to prevent this fatal condition in the future.

**Poster Presentation 4**

## Association of equine squamous and glandular gastric disease with dental status in 54 horses

### R. Lensing^1^, **A. K. Barton** ^1^, F. Thünker^1^, C. Wirth^1^, R. Merle^2^

#### ^1^Equine Clinic Hochmoor, Gescher, Germany; ^2^Freie Universitaet Berlin, Berlin, Germany

**Introduction**: Gastric pH seems to be a key factor for the development of Equine Gastric Ulcer Syndrome (EGUS). It is influenced by amount and duration of roughage uptake, as chewing increases the production of alkaline saliva. The proton‐pump‐inhibitor omeprazole is currently recommended in the ACVIM/ECEIM consensus statement (CS) for therapy of both Equine Squamous Gastric Disease (ESGD) and Equine Glandular Gastric Disease (EGGD). We hypothesized that dental disease decreases saliva production and in consequence gastric pH, therefore predisposing horses to EGUS.

**Material and methods**: Intragastric pH after 12 hours of feed and 3 hours of water withdrawal was documented in 54 horses (September 2023‐April 2024). Scoring included grades 0 to 4 for ESGD (CS), 0 to 3 for EGGD (modified from CS), and 0 to 3 for dental disease. Omeprazole therapy (buffered formulation 4 mg/kg PO SID, Gastrogard, Boehringer Ingelheim or enteric coated granules 2 mg/kg PO SID, Equizol, CP Pharma) was recommended (n = 32), dental disease was corrected (n = 20) and gastroscopy was repeated 4 weeks later (n = 9).

**Results**: The Chi^2^ test showed a trend that dental disease grade 3 to 4 was associated with a low gastric pH (1‐4) (*P* = .100). Moderate‐severe dental disease increased the chance of low gastric pH by factor 2.6 (odds ratio [OR] = 2.57; 95% confidence interval [CI] 0.124‐1.214). An association between dental disease and ESGD (≥2/4) (*P* = .394) was not clearly shown and no association was found for EGGD (≥2/3) (*P* = .857). ESGD was improved ≥2 subgrades or achieving grade 0 in 5/9 of horses and EGGD was improved ≥1 subgrade or achieving grade 0 in 3/9 horses after omeprazole therapy.

**Discussion and clinical relevance**: No influence of dental diseases on ESGD/EGGD could be determined, but may have been found in a larger study population. There is no evidence that gastroscopy should be recommended in horses with dental disease and vice versa.

**Poster Presentation 5**

## Antimicrobial treatment approaches to horses with acute diarrhea admitted to referral institutions

### **D.E. Gomez** ^1^, R.E. Toribio^2^, L. Hostnik ^2^, D. Byrne^3^, J. Kopper^4^, Group. MEDS^1^

#### ^1^University of Guelph, Guelph, Canada; ^2^The Ohio State University, Columbus, Ohio, USA; ^3^Murdoch University, Murdoch, Australia; ^4^Iowa State University, Ames, Iowa, USA

**Introduction**: A multicenter description and comparison of antimicrobial treatment approaches for acute diarrhea in horses is missing. This study aimed to describe and compare the treatment approaches for diarrheic horses used in different regions of the world.

**Study Design**: Multicenter retrospective study.

**Methods**: Information from diarrheic horses presented to participating institutions between 2016 and 2020, including clinicopathological data, pathogens detected, and antimicrobial drug use, was collected.

**Results**: This study included 1438 horses and 26 referral institutions across 5 continents (North and South America, Europe, Australia, and Asia). The fatality rate was 24%, with no differences between regions. A pathogen was identified in 16% of the horses. 55% (792/1419) of the horses were administered one or a combination of antimicrobial drugs within the first 24 hours of admission. Penicillin and gentamicin were the most used combination (25%, 198/792). The proportion of horses treated with antimicrobial drugs differed among institutions, varying from 17% to 94%. The proportion of horses treated with antimicrobial drugs was lower in Europe and Australia than in the other geographic areas (*P* < .05). In total, 540/1139 (47%) horses had leukopenia, and 70% (380/540) of those horses were administered antimicrobial drugs, while 49% (293/599) of the horses without leukopenia received antimicrobial drugs. 66% (794/1105) of the horses met SIRS criteria, and 28% (311/1105) did not. Of those, 68% (542/794) and 44% (136/311) of the horses that did and did not me SIRS criteria, respectively, were administered antimicrobial drugs.

**Discussion**: Treatments varied between regions and hospitals. Prospective clinical trials are required to evaluate the effects of antimicrobial treatment on survival.

**Clinical relevance**: The high prevalence of antimicrobial use in diarrheic horses, even without evidence of systemic compromise, indicates that factors other than disease severity influence clinicians' decisions to administer antimicrobial drugs to diarrheic horses.

**Poster Presentation 6**

## Does equine asthma predispose horses to premature complexes during exercise?

### **H. Carstensen** ^1^, C.B. Larsen^1^, L.H. Kabrit^1^, R. Buhl^1^, L.C. Berg^1^, C. Hopster‐Iversen^1^, J. Fjeldborg^1^, S. Hansen^1^

#### ^1^Faculty of Health and Medical Sciences, University of Copenhagen, Taastrup, Denmark

**Introduction**: A recent study indicated an association between mild‐moderate equine asthma (MEA) and premature complexes (PCs) during exercise in Standardbred racehorses. Furthermore, asthma in humans is known to be associated with increased risk of arrhythmias. The hypothesis of this study was that horses with MEA based on clinical signs and bronchoalveolar lavage (BAL) cytology have increased number of PCs during exercise and recovery.

**Methods**: A total of 59 horses with a history of poor performance were divided into three groups: Warmbloods, Racehorses and Icelandic horses. All horses underwent a clinical examination, an exercise test with ECG and a BAL. MEA was diagnosed based on increased neutrophil (>5%), eosinophil > (>1%), and/or mast cell (>2%) percentages in the right and/or left lung. PCs during exercise and recovery were detected by ECG analysis. Atrial and ventricular PCs were not distinguished.

**Results**: MEA was diagnosed in 83% of the horses. One or more PCs were observed in 31% of the horses during exercise and in 2% during the recovery period, with the majority found among the Racehorses (13/18 horses). A significant association between max heart rate (HR) and the occurrence of PCs was found (*P* < .0001). No significant association was found between MEA and the occurrence of PCs (*P* = .15). A negative correlation was noted between the number of mast cells in BAL from the right side and the occurrence of PCs (*P* = .04).

**Discussion**: An association between MEA and the presence of PCs could not be confirmed in this study. Future studies should include a control group without poor performance, standardized exercise tests and larger group sizes as breed differences and/or max HR may have influenced the results.

**Clinical Relevance**: This study highlights the importance of considering MEA and cardiac arrhythmias as two distinct causes of poor performance that may occur in the same horse.

**Poster Presentation 7**

## Discovering genetic variants linked to exercise‐induced atrial fibrillation in racehorses

### **S.D. Nissen** ^1^, M. Mattingsdal^2^, L.C. Nath^3^, E. Sørensen^4^, C. Fintl^5^, A. Tveit^2^, M. Aaronaes^6^, M. Myrstad^2^, R. Buhl^7^, S. Franklin^3^, I.E. Christophersen^2,8^

#### ^1^University of Copenhagen, Copenhagen, Denmark; ^2^Baerum Hospital Vestre Viken Trust, Gjettum, Norway; ^3^University of Adelaide, School of Animal & Veterinary Sciences, Adelaide, South Australia, Australia; ^4^Oslo University Hospital Ulleval, Department of Cardiology, Oslo, Norway; ^5^Norwegian University of Life Sciences, Department of Companion Animal Clinical Sciences, Oslo, Norway; ^6^Diakonhjemmet Hospital, Department of Internal Medicine, Oslo, Norway; ^7^Faculty of Health and Medical Sciences, University of Copenhagen, Taastrup, Denmark; ^8^Department of Medical Genetics, Oslo University Hospital, Oslo, Norway

**Introduction**: Atrial fibrillation (AF) is a prominent cardiac arrhythmia in human athletes and is frequently observed in equine athletes too. The prevalence of AF in Thoroughbred (TB) and Standardbred (STB) racehorses is notably high (~5%), echoing the heightened risk evident in human athletes, thereby suggesting exercise as a distinct risk factor. Racehorses, benefiting from selective breeding and detailed pedigree management, offer an ideal setting for genetic research. While both common and rare genetic variations contribute significantly to AF, no study has yet pinpointed genetic variants specifically linked to exercise‐induced AF.

**Objective**: We aimed to uncover genetic markers linked to exercise‐induced AF in racehorses through a genome‐wide association study (GWAS).

**Methods**: We collected samples from 181 TB and STB racehorses, including 71 with confirmed paroxysmal or persistent AF. Genomic analysis involved whole‐genome genotyping, imputation of missing markers, and expanding the control group using publicly available data. GWAS was conducted on 7.85 million markers in 71 cases and 296 controls using PLINK2 with covariates adjusted for population stratification, batch and sex.

**Results**: We discovered a notable genetic locus adjacent to the protocadherin gene *PCDH18* linked to AF in horses. This locus has also been linked to the human ECG trait known as P‐wave terminal force. Alterations in P‐wave terminal force are associated with disturbances in atrial conduction and increased atrial dimensions. Additionally, we observed interesting genetic signals related to amino acid transport (*SLC6A15*), myosin binding (*MYBPH*), and myoblast differentiation (*MYF6* and *MYOG*).

**Conclusion**: In this initial analysis, we have uncovered a novel genetic locus linked to atrial function and AF in racehorses. Furthermore, we detected multiple candidate genes warranting further investigation to unravel the genetic underpinnings of exercise‐induced AF.

**Clinical relevance**: Our findings contribute to the creation of risk assessment tools for exercise‐induced AF and the development of novel medications for treatment.

**Poster Presentation 8**

## Exploring the molecular landscape of equine atrial fibrillation: A multiomics study

### **S.L. Haugaard** ^1^, S.D. Nissen^1^, J. B. Armstrong ^2^, M. Schneider^1^, S.A.T. Kjeldsen^1^, H. Carstensen^1^, C. Iversen^1^, A. Altintas^3^, K.M. Herum^4^, T. Jespersen^2^, T.A. Jepps^2^, R. Buhl^1^

#### ^1^Department of Veterinary Clinical Sciences, Taastrup, Denmark; ^2^Cardiac Physiology Laboratory, Copenhagen, Denmark; ^3^Novo Nordisk Foundation Center for Basic Metabolic Research, Copenhagen, Denmark; ^4^Research and Early Development, Måløv, Denmark

**Introduction**: Atrial fibrillation (AF) induces electrical, contractile and structural remodeling of the heart, which are associated with disease progression and treatment resistance. Here, we aimed to characterize the transcriptomic and proteomic changes in the early stages of sustained AF, to better understand the mechanisms contributing to AF induction and maintenance.

**Methods**: We collected biopsies from the right atrium (RA), left atrium (LA), and left ventricle (LV) of eight horses with naturally occurring sustained AF (mean estimated AF duration: 47 ± 36 days) and eight controls. We performed data‐independent acquisition mass spectrometry on all samples and bulk RNA‐sequencing on six samples from each group. Expression profiles were compared between the AF and control group.

**Results**: We identified a total of 3400 proteins, several of which were differentially regulated between AF horses and controls (268 in RA, 324 in LA, and 284 in LV, *P* < .05). Proteomic pathway enrichment analyses revealed changes in metabolic, contractile and extracellular matrix (ECM) proteins. Notably, atrial proteins associated with oxidative phosphorylation were upregulated in AF. With transcriptomics, we detected a total of 38 849 transcripts, of which 1102 (RA), 534 (LA), and 41 (LV) genes were differentially regulated between groups (adj. *P* < .05). Gene‐set enrichment analysis showed upregulation of ECM and endoplasmic reticulum stress response pathways in both atria, while genes related to muscle cell differentiation and orphan nuclear receptor transcription factors (NR4A1, NR4A2) were downregulated. Atrial ECM remodeling was confirmed by histological staining.

**Conclusion**: Our findings detail molecular pathways associated with early AF in horses, which were characterized by substantial ECM remodeling, upregulation of metabolic proteins and transcriptomic evidence of the unfolded protein response.

**Clinical relevance**: The molecular changes observed in the early stages of disease reveal new potential drug targets for mitigating disease progression and underscore the importance of early intervention in horses with AF.

**Poster Presentation 9**

## The TASK‐1 potassium channel in equine atrial myocardium as a potential target to treat atrial fibrillation

### **L.M. Verhaeghe** ^1^, A. Decloedt^1^, I. Vernemmen^1^, E. Buschmann^1^, G. Van Steenkiste^1^, M. Demeyere^1^, A. Paasche^2^, N. Jávorszky^2^, F. Wiedmann^2^, J.C. Schmidt^2^, G. Van Loon^1^

#### ^1^Ghent University, Gent, Belgium; ^2^Heidelberg University, Heidelberg, Germany

**Introduction**: The TASK‐1 potassium channel is a potential target for novel atrial fibrillation therapies. This study aimed to compare human and equine TASK‐1 channels and to confirm the presence and atrial‐specific expression of TASK‐1 channels in equine myocardial tissue. The effect of a TASK‐1 blocker, ketodoxapram, was evaluated in vivo.

**Methods**: Reciprocal Basic Local Alignment Search Tool analysis identified equine orthologues of human TASK‐1 genes in the horse genome. Using the established *X. laevis* oocyte system and two‐electrode voltage clamp electrophysiology, the functional properties of TASK‐1 channels were investigated. Myocardial tissue samples were collected in 12 horses euthanized for non‐cardiovascular reasons. TASK‐1 expression was analyzed using the StepOnePlus PCR system, with RNA quantification and integrity assessed by spectrophotometry and gel electrophoresis. Prior to euthanasia, 0.5 mg/kg ketodoxapram was administered intravenously to six horses under general anesthesia. Measurements before and after ketodoxapram included atrial effective refractory period (AERP) via intracardiac catheterization, blood pressure, respiratory rate, heart rate and P wave duration.

**Results**: Human and equine TASK‐1 channel isoforms are highly homologous, sharing DNA sequences with 96.5% identity and 97.5% similarity at protein level. Functional similarity could be observed as equine TASK‐1 channels had the same current‐voltage relationship as the human isoforms. Equine TASK‐1 channel expression was higher in the atria compared to the ventricles (*P* = .0023). Ketodoxapram administration resulted in increased AERP (median 250 [210‐340] ms vs 420 [290‐530] ms, *P* = .042) with transient increases in blood pressure and respiratory rate, without changes in heart rate or P wave duration.

**Conclusion**: There is a high degree of structural and functional similarity between human and equine TASK‐1 channels, with predominantly atrial expression. AERP increased significantly after ketodoxapram administration without adverse effects.

**Clinical relevance**: Medical treatment options for AF horses remain limited. Ketodoxapram, a TASK‐1 blocker, might be a promising, safe drug for treating equine AF.

**Poster Presentation 10**

## Preliminary validation of a smart textile device during high‐speed exercise for ECG quality and heart rate measurement

### **A. Kapusniak** ^1^, S. Franklin^1^, P.L. Hitchens^2^, S. Bailey^2^, P. Mccrae^3^

#### ^1^School of Animal and Veterinary Sciences, University of Adelaide, Roseworthy, Australia; ^2^University of Melbourne, Melbourne, Australia; ^3^Myant Inc., Toronto, Canada

**Introduction**: Widespread assessment of exercise‐induced arrhythmias in horses requires user‐friendly cardiac monitors. This study aimed to evaluate a novel smart textile band (Skiin Equine; Myant Inc.) for recording of electrocardiograms (ECGs) during strenuous exercise by comparing diagnostic quality and measurements of heart rate with a reference ECG device.

**Methods**: ECGs were recorded simultaneously using the Televet II and Skiin Equine device in 16 separate exercise tests on 13 healthy Standardbred racehorses in training, during high‐speed exercise. GPS data was extracted to determine maximum speed for each trial. ECGs were evaluated using Kubios software, where the percentage of artifact was recorded, and the diagnostic quality of the traces assessed by a blinded observer. Maximal heart rate (HR_max_; calculated over 30 seconds) was calculated over 30 seconds during peak exercise and compared between devices. Pearson's correlation was performed and Bland‐Altman test used to assess agreement (bias, 95% limits) between the two devices.

**Results**: The median (IQR) peak speed during exercise was 13.90 m/s (13.15‐14.25 m/s). One Skiin ECG and 2 Televet ECGs had artifact >10%. One Televet trace was excluded from further analysis due to loss of an electrode during maximal exercise. Mean (SD) HR_max_ was 225 bpm (4.9) for Skiin vs 223 bpm (4.9) for Televet. There was a very strong correlation between devices (*r* = .94; *P* < .01) and agreement was 1.53 bpm (−3.4, 3.4).

**Conclusion**: The Skiin Equine device provides a reliable ECG trace for heart rate detection, potentially allowing its application to widespread monitoring of horses during high‐speed exercise. Further study is required to confirm its diagnostic capabilities for arrhythmia detection.

**Clinical Relevance**: New smart textile technology may play a valuable role in cardiac arrhythmia detection and diagnosis.

**Poster Presentation 11**

## Intra and interobserver reliability of pulsed‐wave tissue Doppler imaging of the left ventricle in healthy Standardbred neonatal foals

### **F. Timbó D'el Rey Dantas** ^1^, A. Mannini^1^, C. Castagnetti^1^, L. Menchetti^2^, G. Romito^1^, V. Cevoli^1^, F. Freccero^1^

#### ^1^Università di Bologna, Ozzano Dell'Emilia, Italy; ^2^Università di Camerino, Matelica, Italy

**Introduction**: Little is known about tissue Doppler imaging (TDI) of the left ventricle (LV) in foals. The aim of this study was to assess intra and interobserver reliability of pulsed‐wave (PW) TDI measurements for characterization of LV wall motion in healthy foals.

**Methods**: Transthoracic echocardiography was performed in seven healthy Standardbred foals within 24 hours of life by an experienced operator. PW‐TDI tracings were acquired on the LV free wall (LVFW) at the level of the chordae tendinae from a right parasternal short axis view, and on the septal and parietal LV segments at the level of the mitral annulus from a subcostal (SC) view to assess radial and longitudinal LV velocity profiles. PW‐TDI systolic (S_1_, peak velocity during isovolumic contraction; S_m_, peak systolic velocity) and diastolic (E_1_ peak velocity during isovolumic relaxation; E_m_, peak proto‐diastolic velocity; A_m_, peak late diastolic velocity) velocities were measured from the three views by four independent observers with various experience (equine internal medicine board‐certified, PhD student, junior clinician, undergraduate student). Intra and interobserver reliability were evaluated using intraclass correlation coefficients (ICC). Measurement variability was quantified calculating the within‐subject coefficient of variation (CV) and repeatability coefficient (RC).

**Results**: Interobserver agreement was excellent (ICCs >0.75) for all variables except S_m_ and E_m_ at the septal LV segment which showed a good agreement (0.60 ≤ ICC < 0.75). Intraobserver reliability was good or excellent, except for S_m_ at the septal LV segment which was poor. The CV was <10% for all radial motion variables measured at the LVFW and longitudinal motion variables at the parietal but not septal LV segment.

**Conclusions**: PW‐TDI indices of LVFW radial and longitudinal wall motion have excellent inter and intraobserver reliability in healthy neonatal foals.

**Clinical relevance**: PW‐TDI indices may be implemented in the assessment of LV function of neonatal foals.

**Poster Presentation 12**

## Bicuspid pulmonary valve in horses: A case series

### **E. De Bruijn** ^1^, E. Paulussen^1^, A. Dufourni^1^, G. van Loon^1^

#### ^1^Internal Medicine University Ghent, Merelbeke, Belgium

**Introduction**: Bicuspid pulmonary valve (BPV) is a rare congenital condition described in human medicine, often associated with other congenital heart diseases. The occurrence of BVP is rarely mentioned in equine literature.

**Methods**: Retrospective data (8 year period) were reviewed of all horses admitted for cardiac evaluation (n = 2115) because of a murmur, arrhythmia, poor performance or congestive heart failure.

**Results**: BPV was echocardiographically identified from a left‐sided parasternal short‐axis view in 19 horses. Median age was 4 years (range 8 days to 22 years). Ten horses showed BVP combined with a ventricular septal defect (VSD), of which four showed additional congenital cardiac anomalies. In five, moderate to severe pulmonary valve regurgitation was present. Nine showed some degree of pulmonary stenosis, of which three had a “hockey‐stick” pulmonary valve, and three moderate stenosis (peak flow velocity >2.6 m/s). One horse showed a bilateral holodiastolic (2/6) and left‐sided holosystolic (5/6) murmur correlated with moderate pulmonary regurgitation and the hockey‐stick valve conformation, respectively. In the other horses, attributing specific murmurs to the presence of a BPV was challenging due to coexistence of other heart diseases. In three horses marked pulmonary artery dilatation was present, of which one horse with also moderate pulmonary valve regurgitation was retired, and of which 2 had to be euthanized due to additional congenital heart disease and signs of congestive heart failure.

**Conclusion**: BPV was often associated with VSD or other valvular regurgitation. In many patients BPV was thought to have no or limited clinical effects at the moment of examination. In three patients BPV resulted in severe pulmonary valve regurgitation, stenosis or pulmonary artery dilatation.

**Clinical relevance**: Left parasternal views of pulmonary valve and artery allow to diagnose BPV, pulmonary stenosis, presence of a hockey‐stick valve and pulmonary artery dilatation, and should be included in routine echocardiography.

**Poster Presentation 13**

## Equid hepatitis b virus detected in two livers of donkeys with hyperlipemia

### **V. Zehetner** ^1^, J.M. Cavalleri^1^, I. Preining^1^, A. Klang^2^, M. Hofer^3^, A.S. Ramsauer^1,4^

#### ^1^Equine Internal Medicine, University of Veterinary Medicine Vienna, Vienna, Austria; ^2^Institute of Pathology, University of Veterinary Medicine, Vienna, Austria; ^3^Shared Facilities, VetCore, University of Veterinary Medicine, Vienna, Austria; ^4^Institute of Virology, Vetsuisse Faculty, University of Zurich, Zurich, Switzerland

**Introduction**: Recent research has revealed significant similarities between the newly detected equid hepatitis B virus (EqHBV) and the hepatitis B virus (HBV) in humans. EqHBV was detected in 3.2% of sampled donkeys and zebras with a seroprevalence of 5.4% and displays a worldwide distribution. So far, all horses have tested negative. In donkeys, the virus exhibits hepatotropism and can lead to chronic infections, with histopathologic findings such as inflammation and fibrosis resembling those seen in HBV infections. This study aims to determine the prevalence and impact of EqHBV on different equid hepatopathies.

**Methods**: A retrospective analysis was conducted on 92 formalin‐fixed, paraffin‐embedded (FFPE) liver samples, including 79 horses and 5 donkeys diagnosed with various hepatopathies and 8 healthy control livers. FFPE sections were stained with Hematoxylin and Eosin for histopathologic evaluation. DNA was extracted from FFPE liver samples and quantitative PCR (qPCR) analysis was performed to detect the presence of EqHBV‐DNA. Positive samples underwent digital PCR (dPCR) to quantify viral loads.

**Results**: Two livers tested positive for EqHBV‐DNA by qPCR. Both originated from donkeys clinically diagnosed with hyperlipemia. Viral load measured by dPCR was 12.25 × 10^3^ GE/million cells in case 1 and could be detected but not reliably quantified in case 2. Histologic examination revealed severe hydropic degeneration of hepatocytes and mild focal portal inflammation (case 1), respectively moderate diffuse lipidosis and multifocal portal/periportal fibrosis (case 2).

**Discussion**: EqHBV could be detected in two of five livers of donkeys diagnosed with hyperlipemia, whereas all livers from horses were negative. Due to the low measured viral loads and unspecific histopathological findings, subclinical viral persistence may be possible, but further studies are required to better characterize the clinical relevance of EqHBV infections in donkeys.

**Clinical relevance**: This study provides further evidence for the occurrence of EqHBV in donkeys as major host.

**Poster Presentation 14**

## Clinical presentation, treatment and outcome of foals with umbilical infections (2014‐2023)

### **C. Ribonnet** ^1^, V. Colgate^1^, E. Floyd^1^

#### ^1^Rossdales Veterinary Surgeons, Newmarket, United Kingdom

**Introduction**: Umbilical infections are common and important in foals. There are no recent reports describing clinical presentation, microbiology and management of foals with umbilical infections in the United Kingdom.

**Methods**: Retrospective review of case records of foals with clinical and/or ultrasonographic evidence of umbilical infection between 2014 and 2023. Data were analyzed using Mann‐Whitney *U* and chi squared analysis.

**Results**: Exactly 118 records were included, 34 foals had umbilical disease alone, 36 had non‐infectious comorbidities and 48 had other infection sites (7 gastro‐intestinal, 4 respiratory, 11 musculoskeletal and 26 multiple comorbidities). Short‐term survival was 92% (95% CI 87%‐97%). Median antimicrobial therapy length was 18 days (IQR 13‐26), 108 foals were treated conservatively and 10 had surgical resection of the umbilicus.

The most common isolates of all culture samples were *E. coli* (20%), beta‐hemolytic *Streptococci* (18%), *Enterococcus* sp. (12%) and of only umbilical samples were beta‐hemolytic *Streptococci* (31%), *E. coli* (23%), *S. aureus* (14%). Seven foals had multi‐resistant bacteria cultured, one underwent surgery and all survived. Data from all samples showed non‐protected antimicrobials with the highest sensitivity for gram‐positive bacteria were rifampicin (78%, 95% CI 69%‐88%), clarithromycin (68%, 95% CI 57%‐78%), azithromycin (65%, 95% CI 54%‐76%), and for gram‐negative bacteria, amikacin (93%, 95% CI 85‐100%), gentamycin (69%, 95% CI 55%‐83%), doxycycline (67%, 95% CI 52%‐80%). In foals with infectious comorbidities, median fibrinogen and SAA were higher (6 vs 4.1; 400 vs 2; *P* < .001), antimicrobial therapy shorter (16 vs 19; *P* = .02), hospitalization longer (8 vs 3; *P* < .001) and survival lower (100% vs 83%; *P* < .001); there was no difference in medical treatment or surgical resection of the umbilicus (6% vs 10%; *P* = .47).

**Discussion**: Foals with umbilical infections often have multiple site infections. Survival rates are high, but foals can require a prolonged course of antimicrobials.

**Clinical Relevance**: This study provides information to help veterinarians with evidence‐based recognition and treatment of umbilical infections.

**Poster Presentation 15**

## High prevalence of subclinical equid hepatitis b virus infection in a donkey farm in Romania

### **D. Lale** ^1^, A.N. Mureşan^2^, A.S. Anna Sophie^1^

#### ^1^Institute of Virology, Vetsuisse Faculty, University of Zurich, Zurich, Switzerland; ^2^Department of Internal Medicine, Faculty of Veterinary Medicine, UASVM, Cluj‐Napoca, Romania

**Introduction**: Equid viral hepatopathies caused by equine hepacivirus (EqHV), equine parvovirus‐hepatitis (EqPV‐H), and the recently first‐time described equid hepatitis B virus (EqHBV) are poorly understood in donkeys. Only one recent study reported the occurrence of EqHBV with a prevalence of 3.2% (29/906) in donkeys and zebras. Similar to infection with hepatitis B virus (HBV) in humans, EqHBV infection in donkeys leads to hepatotropism, chronic infections, and possible simultaneous infection with other hepatic viruses. The study aimed to investigate the occurrence of equid hepatic viruses in an isolated donkey herd and look for possible co‐infections.

**Methods**: In this pilot study, 100 serum samples collected for routine diagnostics from a donkey herd used for milk production in northwest Romania were analyzed for the presence of EqHBV‐DNA, EqPV‐H‐DNA by quantitative PCR (qPCR), and EqHV‐RNA by RT‐qPCR.

**Results**: Of the 100 collected serum samples, 32% tested positive for EqHBV‐DNA, while EqHV‐RNA and EqPV‐H‐DNA could not be detected. The donkey herd consisted of 76 jennies and 24 jacks, which showed no abnormalities during routine clinical examination.

**Discussion**: Subclinical infection with the recently described EqHBV was detected in a clinically healthy donkey herd. The prevalence of viremia in this cohort was higher than previously reported. No coinfections with EqHV or EqPV‐H were detected.

**Clinical relevance**: As HBV can cause serious liver disease in humans, the detection and further characterization of EqHBV infections and potential coinfections with other hepatic viruses in donkeys are essential to determine their clinical relevance and improve the health of donkeys.

**Poster Presentation 16**

## Prevalence of strongylus vulgaris in danish horses and treatment response

### K.S. Meilvang^2^, **J.C. Borrye** ^2^, T.H. Pihl^1^, I. Bartholdy^2^, K. Kristensen^2^, C. Rendtorff^2^, M. Hjortflod^2^, S.M. Thamsborg^3^, M.K. Nielsen^4^, K. Nielsen^2^

#### ^1^Department of Veterinary Clinical Sciences, Faculty of Health and Medical Sciences, Copenhagen, Denmark; ^2^Department of Veterinary Clinical Sciences, Faculty of Health and Medical Sciences, Copenhagen, Denmark; ^3^Department of Veterinary and Animal Sciences, University of Copenhagen, Copenhagen, Denmark; ^4^ The Maxwell H. Glucks Equine Research Center, University of Kentucky, Lexington, USA

**Introduction**: The prevalence of *S. vulgaris* is increasing in Denmark and Sweden, and several studies indicate that selective therapy based only on fecal egg counts are contributing to this development. Evidence‐based treatment guidelines for equine herds diagnosed with *S. vulgaris* are frequently requested by equine veterinary practitioners, but studies evaluating the efficacy of different treatment strategies have never been performed. Estimation of *S. vulgaris* prevalence in equine herds in Zealand (DK) and evaluation of two treatment protocols in infected herds: a single ivermectin treatment and two ivermectin treatments given 6‐weeks apart.

**Methods**: The prevalence of *S. vulgaris* was estimated with both larval culture and qPCR on isolated eggs, and these results were compared by McNemar's test. Herds identified as positive for *S. vulgaris* in larval culture (one or more positive horses) were divided into two groups receiving two different treatments. Follow‐up fecal samples were evaluated at week 8 and 24 post initial treatment.

**Results**: In total, 299 horses from 30 herds were included in the study. The prevalence estimated by qPCR was 5.74% (95% CI: 3.45%‐9.21%) on individual horse level and 33.33% (95% CI: 17.94%‐52.87%) on herd level. The prevalence estimated by larval culture (2.68%, 95% CI: 1.25%‐5.41%) was significantly lower (*P* = .04) than the prevalence estimated by qPCR. Both treatment protocols appeared equally effective, as all follow‐up samples were negative for *S. vulgaris*.

**Clinical relevance**: Prevalence of *S. vulgaris* was lower than expected and might be explained by the participating veterinarians already having focus on testing for *S. vulgaris* with yearly larval cultures and treatments of all horses on the farm when finding one positive horse. One treatment with ivermectin seems to be effective, but further studies with more positive horses need to be performed.

**Poster Presentation 17**

## A retrospective study on intradermal allergy test (IDT) results and efficacy of allergen specific immunotherapy (ASIT) in horses with equine asthma (EA), equine allergic skin disease (EASD) and head shaking (HS)

### **M. Welters** ^1^, S.T. Auxilia^1^, K. Shell^2^, N. Meyer^2^

#### ^1^Anicura Haaglanden, Rijswijk, Netherlands; ^2^Pferdeinternistik, Leichlingen, Germany

**Introduction**: IDT was performed in 84 privately owned horses affected by EA, EASD, and/ or HS. A total of 55 received ASIT based on IDT results. The triggering allergens for each horse were identified and ASIT efficacy was assessed.

**Methods**: A retrospective study was conducted on 84 horses diagnosed with EASD, HS or EA with indication of an allergic component, such as eosinophilia in lung secretion and/or seasonality. Diagnostics were performed based on the most recent consensus guidelines. Seven groups were made: EA (22/84), EASD (24/84), and HS (6/84), EA with EASD (20/84), EA with HS (1/84), EASD with HS (2/84), and 9 cases with mixed symptoms.

IDT was performed in all horses including up to 23 area and species relevant allergens. Results were evaluated at 30 minutes (by veterinarian), 3 and 24 hours, by the veterinarian or the owner.

Efficacy of ASIT was evaluated by a standardized telephone questionnaire (Stepnik et al, Veterinary Dermatology, 23, 29‐e7, 2011) after at least 8 months of therapy.

**Results**: Exactly 84 horses tested positive on IDT and 55 were treated with ASIT, while 29 due to owners preferences or incompatible results were not. Among the 55, 32 (58%), were followed up with a questionnaire, including 9 EA (16%), 10 EASD (18%), 3 HS (10%) and 10 combined EA‐EASD (18%) cases.

Response to ASIT based on owners' perception:

EA: 6/8 good to excellent, 2/8 no improvement.

EASD: 7/8 good to excellent, 1/8 no improvement.

HS: 2/3 good to excellent, 1/3 no improvement.

Combined EA‐EASD 7/7 good.

**Conclusions**: Using a multimodal therapeutic approach, ASIT based on IDT can be beneficial in treating patients with EA and HS, as known for EASD. Further studies ideally within a controlled environment, with regular yearly follow up evaluations by clinicians and via questionnaires are necessary to confirm the efficacy of this therapy.

**Poster Presentation 18**

## Prevalence of catheter‐associated thrombophlebitis in horses at a veterinary teaching hospital from 2012 to 2022

### S. Fauquet^1^, **M. Allano** ^1^, T. Juette^1^, L. Kamus^1^

#### ^1^Université de Montréal, Saint‐Hyacinthe, Canada

**Introduction**: In veterinary clinics, thrombophlebitis (TB) is the most common complication following intravenous (IV) catheterization. Risk factors have been described in horses, but there is little data on prevalence in hospitals. The objectives of this study were to estimate the prevalence of IV catheter‐associated TB in an equine hospital and to describe temporal variations over 10 years.

**Methods**: Equids hospitalized for at least 1 day from 2012 to 2022, with thrombophlebitis (n = 310) and, with IV catheters (n = 4490) were identified retrospectively through medical records. TB cases not related to IV catheterization were excluded. Signalment, date of admission, and duration of hospitalization were collected. The effect of variables (age, gender, breed, year, month, season, duration of hospitalization) on the prevalence of TB was evaluated through generalized linear mixed models (R software).

**Results**: The patients with TB presented a mean age of 8.5 years (median 8.0 [0‐29.9]), were mostly Quarter Horses, and had a mean hospital stay of 17.7 days (median 13 [1‐110]). The prevalence of TB between 2012 and 2022 was 6.9% (95% CI: 6.1‐7.6). Significantly higher prevalence was observed in 2013, 2021, and 2022 (*P* < .05). In 2022, TB were more prevalent during winter (*P* < .04). There is a statistical effect of the hospitalization duration on TB prevalence. The patients with TB were hospitalized 17.7 days (95% CI: 17.3‐18.1), compared to those without TB, 9.8 days (95% CI: 8.1‐11.5).

**Conclusions and clinical relevance**: The prevalence of IV catheter‐associated thrombophlebitis at the CHUV did not increase over 10 years, except for 2021 and 2022, and seems not to be influenced by seasons. Identifying trends and basal rates in a veterinary hospital is important to detect variations outside the normal range. The variables studied, and possible risk factors, are directly affected by the quality of data extraction.

**Poster Presentation 19**

## Cystic calculi in horses: A retrospective study of 47 cases (2006‐2023)

### A.R. Rousseau^1^, **D.J. Jean** ^1^, L.K. Kamus^1^

#### ^1^University of Montreal, St‐Hyacinthe, Canada

**Introduction**: Large clinical studies of cystic calculi in horses are limited.

**Objectives**: Describe the clinical findings, alterations in renal function and short‐term survival rate of horses with cystic calculi.

**Methods**: Medical files of equids with cystic calculi presented to the Equine Hospital at University of Montreal (2006‐2023) were reviewed. Extracted information included: patient data, reason for admission, clinical exam findings, blood workup, urinary tract procedures and treatments. Follow‐up was obtained over the phone with the owners.

**Results**: Forty‐seven equids (24 gelding, 22 females and one stallion) with a wide age range (1‐25 years) met the inclusion criteria including two patients treated for recurrence (n = 49 hospitalized cases). Cystic calculi were identified in 0.24% of all patients examined in our equine hospital in internal medicine and surgery services. Quarter Horse (31.9%) and Standardbred (19.1%) were overrepresented compared to our equine hospital population. Hematuria (34/49 hospitalized cases) was the most frequent clinical observation in these equids. The short term survival rate was 96% (45/47 cases) and two horses were euthanized after recurrence of cystic calculi for financial and prognosis reasons. Hypercreatinemia was observed in 17/38 cases (44.7%) and most cases (13/17) had slight increase of creatinine (between 135 and 200 μmol/L; normal range between 70 and 134 μmol/L) throughout their hospitalization. Perineal urethrotomy was mostly performed in males (22/27 hospitalized cases) and the transurethral approach was most frequently used in females (15/22 hospitalized cases). No significant differences were observed between the type of anesthesia (standing vs general anesthesia) and the length of hospitalization.

**Discussion**: The prevalence of cystic calculi is similar than previously reported and females were more frequently involved. Little impact on renal function was observed in our patients.

**Clinical relevance**: Patients with cystic calculi in this study showed a favorable prognosis and choice of surgical procedure depending of the sex of equids.

**Poster Presentation 20**

## The role of oxidative stress in the equine asthma pathogenesis

### **S. Hansen** ^1^, C.P. Rubio^2^, J.J. Jose^2^

#### ^1^University of Copenhagen, Taastrup, Denmark; ^2^University of Murcia, Murcia, Spain

**Introduction**: Inhalation of environmental allergens causes airway inflammation and the production of reactive oxygen species. Superoxide dismutase (SOD), an enzymatic antioxidant can partly counterbalance oxidative stress. The aim of this study was to investigate the local concentration of SOD in the lung fluid of horses with equine asthma (EA).

**Methods**: A commercially available spectrophotometric assay (Randox LTD) was validated for the measurements of SOD in tracheal wash (TW) and bronchoalveolar lavage (BAL) samples. Then SOD was measured in these fluids in a group of healthy horses (n = 37) as well as horses with varying degree of neutrophilic EA (neutrophilic mild‐moderate EA [n = 29] and severe EA [n = 25]).

**Results**: A significant decrease in the concentration of SOD in the BAL fluid was found in horses with mild‐moderate neutrophilic EA and severe EA compared to healthy horses. Further, horses with severe EA had lower values of SOD in TW compared to healthy ones.

**Conclusion**: Results from this study confirm the involvement of oxidative stress and more specific a deficient amount of SOD in the BAL fluid as part of the EA pathogenesis in horses.

**Clinical relevance**: The impairment of SOD in the BAL fluid increased with EA severity. In human studies, low SOD status in asthmatic patients are linked to airway hyperreactivity and airflow obstruction. Future studies should explore a possible beneficial effect of antioxidant supplementation in alleviating clinical signs in asthmatic horses.

**Poster Presentation 21**

## A retrospective study of the incidence and associated risk factors of exertional heat illness (EHI) in thoroughbred racehorses in different states in Australia

### **E.J.M.M. Verdegaal** ^1^, C.J.G. Delesalle^2^, T.J. McWhorter^1^ , G.S. Howarth^1^, H.K. Kennedy^1^

#### ^1^University of Adelaide, Roseworthy, Australia; ^2^Ghent University, Ghent, Belgium

**Introduction**: Heat stress causes suboptimal performance and has significant impact on equine sport disciplines, especially the racing industry. If heat stress progresses to Exertional Heat Illness (EHI), it may become fatal. The incidence of EHI will further increase considering global warming. The main objective of this preliminary retrospective study was to review EHI cases in racehorses.

**Methods**: Data were obtained from online steward reports of Racing Authorities Australia. Racetrack variables, horse‐related variables, environment‐related variables, and heat‐stress case data were evaluated to determine risk factors associated with EHI and divided into two groups: identified EHI cases (1) and suspected EHI cases based on inclusion criteria (2). A descriptive analysis was performed.

**Results**: Across the 2019 to 2020 racing season, 6648 races were evaluated across 151 clubs in four states with a total of 61 064 flat race starters. The EHI incidence per starter (per race) was 0.158% (n = 5 suspected, 1.26%) in Northern Territory (NT), 0.049% (n = 1 and n = 1 suspected, 0.43%) in Tasmania (TAS), 0.039% (n = 2 and n = 3 suspected, 0.36%) in South‐Australia (SA) and 0.054% (n = 20 and n = 2 suspected, 0.50%) in Queensland (QLD). Queensland recorded the highest number in November (n = 7) and January (n = 4). An EHI history was recorded in four, seven, two and one cases in NT, TAS, SA, and QLD, respectively. Poor recovery cases recorded 31 (0.979%), 42 (1.025%), 13 (0.094%), and 96 (0.234%) in NT, TAS, SA, and QLD with an overall incidence of 0.298%. Synchronic diaphragmatic flutter was recorded in none, 4 (0.126%), 18 (0.141%), and 16 (0.039%) racers in NT, TAS, SA, and QLD, respectively, overall, 0.062% and was never associated with EHI.

**Discussion**: The reported overall incidence of 0.056% indicates variances in diagnosing EHI across states, showing potential gaps in veterinarian, steward and rider awareness of EHI.

**Clinical Relevance**: Continued research into risk factors is required to prevent EHI in racehorses globally and address this welfare issue.

**Poster Presentation 22**

## Retrospective study of congenital anomalies observed at necropsy from 2015 to 2023

### **P.M. Moreau** ^1^, S.T. Taleb^2^, L.B. Baudet ^1^, A.M. Merlin^1^, J.C. Vallé‐Casuso^1^, N.F. Foucher^1^, M.B. Bernez‐Romand^1^

#### ^1^Anses, Normandy Laboratory for Animal Health, PhEED Unit, Goustranville, France; ^2^Faculty of Medicine, University of Limoges, Limoges, France

**Introduction**: The French surveillance network of equine mortality (RESUMEQ) was created in 2015 for the qualitative monitoring of equine mortality through the centralization of necropsy data in a national database. Congenital malformations, although rare, represent an economic loss for the equine industry. Studies on congenital anomalies are generally limited to abortions or the neonatal period, or concern a particular malformation. The objective was to describe all congenital malformations observed at necropsy.

**Methods**: A retrospective study was performed using necropsy data recorded in the RESUMEQ database from 2015 to 2023. The population consisted of all autopsied equids ranging from fetuses to adults. The malformations were classified according to the anatomical system of malformation and the type of anomalies. Variables were analyzed by logistic regression.

**Results**: A congenital malformation was determined in 49 cases among 2210 equine necropsies (2.2%). All age groups were represented (mean 18 months, median 4 months), however malformations are less common in adults >2 years old (*P* < .01). The age vary depending on the type of malformation; for example, arthrogryposis affects newborn foals, while compressive cervical myelopathy affects young horses. The musculoskeletal system is most frequently affected (37%) with compressive cervical myelopathy, arthrogryposis/scoliosis or osteochondrosis; followed by malformations of the head and/or brain (16%), digestive system (10%), cardiovascular system (8%), multiple systems (8%), and miscellaneous (20%).

**Conclusion**: The prevalence of congenital anomalies at necropsy observed in our study corresponds to those described in the literature although the distribution of malformations is a little different. It was difficult to group the malformations together because some cases had their own particularities, consequently the number of malformations and categories being low, it was not possible to carry out fine statistical analysis.

**Clinical relevance**: This study provides a view of the epidemiology and type of congenital malformations observed at necropsy.

**Poster Presentation 23**

## Selection of acute observation timepoints with intradermal skin testing in horses

### **L.M. Hunyadi** ^1,2^, S.C. Hyde^1^, E.A. Sundman^1,2^

#### ^1^Texas Tech University School of Veterinary Medicine, Amarillo, Texas USA; ^2^Advanced Equine Diagnostics, Kapaau, Hawaii, USA

**Introduction**: Intradermal skin testing (IDT) in horses is a complicated and time‐consuming procedure in ambulatory settings. Few recommendations currently are available for the selection of acute timepoints to evaluate. The objective was to identify the optimum time for acute observations in ambulatory settings.

**Materials and Methods**: Seventeen client‐owned horses in Texas were included in the study. All horses were injected with 23 allergens, 1 positive control (histamine), and 1 negative control (saline). Lesions sides were measured by the maximum diameter 15 and 30 minutes post‐injection. Saline ratio, histamine ratio, and severity were calculated for each lesion.

**Results**: Statistically significant differences in diameter were identified in 13/23 allergens between the two timepoints; however, no statistically significant differences were found in for the saline ratio, histamine ratio, or severity (normal, mild, severe). A total of 5/17 horses had a minimum of 4/23 (17%) positive allergens at 15 minutes that were normal by 30 minutes. Exactly 8/17 horses had a minimum of 4/23 (17%) normal allergens at 15 minutes that were positive by 30 minutes. a total of 14 horses had severe reactions at 15 and 30 minutes for at least one allergen. One horse had no severe reaction at 15 minutes and eight severe reactions at 30 minutes, while two horses had a severe reaction at 15 minutes and no severe reactions at 30 minutes.

**Discussion**: Minor differences were detected in the allergic response to specific allergens between 15‐ and 30‐minute timepoints. Three horses had a different diagnosis of severe allergy between 15‐ and 30‐minute timepoints.

**Clinical Relevance**: Further work is necessary to identify the relevance of temporal changes in lesion size when evaluating acute IDT results and the relationship to delayed IDT results. Evaluation of multiple timepoints may be useful for identifying positive allergens.

**Poster Presentation 24**

## Are the clinical effects of mesenchymal stem cells merely mediated by phagocytosing macrophages?

### **S.F. Chaimbeul** ^1^, J. Bagge^2^, L.C. Berg^1^, G. Cuevas‐Ramos^1^, C. Lindegaard^1^

#### ^1^University of Copenhagen, Taastrup, Denmark; ^2^Sports Orthopedic Research Center, Hvidovre Hospital, Denmark

**Introduction**: Despite the increasing use of intra‐articular injections with mesenchymal stem cell (MSC) in equine joint diseases, the precise mechanisms remain unclear. The synovium, rich in macrophages, can interact with and phagocytose MSCs and this process may trigger changes in macrophage phenotype. The therapeutic benefits of MSCs may arise from their clearance by phagocytic macrophages prompting pro‐resolving responses within the joint.

**Objectives**: The aim of this study is to compare transcriptional and protein profile changes in equine bone marrow‐derived macrophages (BM‐Mϕ) after exposure to allogeneic live or dead adipose derived MSCs (AT‐MSCs), naive BM‐Mϕ, and BM‐Mϕ exposed to *E. coli*.

**Methods**: BM‐Mϕ were isolated from three horses and cultured as per standard protocols. Pooled AT‐MSCs from four horses were used to stimulate. BM‐Mϕ were exposed to either allogeneic live or dead AT‐MSCs or E. coli bioparticles for 3 hours. Dead AT‐MSCs were prepared by a freeze‐thaw cycle to simulate clinical conditions. Cultures were assessed microscopically and then harvested for RNA‐sequencing, flow cytometry and mass‐spectrometry proteomic analysis.

**Results**: Microscopy and flow cytometry analysis confirmed the phagocytic capacity of BM‐Mϕ. RNA‐sequencing revealed significant transcriptomic differences between treatments and naive BM‐Mϕ, with dead AT‐MSCs‐exposed cells displaying the most similar expression profile to the naive BM‐Mϕ. Proteomic results are pending.

**Conclusions**: This study indicates that phagocytosing MSCs affects macrophages when compared to their naive state or when phagocytosing bacteria. Macrophage phagocytosis may contribute, at least in part, to the therapeutic effect of MSC treatment.

**Clinical relevance**: It is crucial to establish whether the therapeutic effect of MSC treatment results from live MSCs or merely from their uptake by macrophages, a mechanism that could potentially be achieved using any cellular matter. It is also highly relevant to understand the effect dead MSC may have, since a percentage of MSCs likely die before or during treatment.

